# A Systematic Review of AI-Based Techniques for Automated Waste Classification

**DOI:** 10.3390/s25103181

**Published:** 2025-05-18

**Authors:** Farnaz Fotovvatikhah, Ismail Ahmedy, Rafidah Md Noor, Muhammad Umair Munir

**Affiliations:** Faculty of Computer Science and Information Technology, Universiti Malaya, Kuala Lumpur 50603, Malaysia; f_fotovati@siswa.um.edu.my (F.F.); fidah@um.edu.my (R.M.N.); chumair17@gmail.com (M.U.M.)

**Keywords:** waste classification, machine learning (ML), deep learning, datasets for waste classification, environmental sustainability, smart waste management

## Abstract

Waste classification is a critical step in waste management that is time-consuming and necessitates automation to replace traditional approaches. Recently, machine learning (ML) and deep learning (DL) have gained attention from researchers seeking to automate waste classification by providing alternative computational techniques to address various waste-related challenges. Significant research on waste classification has emerged in recent years, reflecting the growing focus on this domain. This systematic literature review (SLR) explores the role of artificial intelligence (AI), particularly machine learning (ML) and deep learning (DL), in automating waste classification. Using Kitchenham’s and PRISMA guidelines, we analyze over 97 studies, categorizing AI-based techniques into ML-based, DL-based, and hybrid models. We further present an in-depth review of over fifteen publicly available waste classification datasets, highlighting key limitations such as dataset imbalance, real-world variability, and standardization issues. Our analysis reveals that deep learning and hybrid approaches dominate the current research landscape, with CNN-based architecture and transfer learning techniques showing particularly promising results. To guide future advancements, this study also proposes a structured roadmap that organizes challenges and opportunities into short-, mid-, and long-term priorities. The roadmap integrates insights on model accuracy, system efficiency, and sustainability goals to support the practical deployment of AI-powered waste classification systems. This work provides researchers with a comprehensive understanding of the state-of-the-art in ML and DL for waste classification and offers insights into areas that remain unexplored.

## 1. Introduction

Waste management is becoming an increasingly critical global challenge due to rapid urbanization, population growth, and economic development. According to the World Bank, global waste generation is projected to increase dramatically and reach up to 3.4 billion tons by 2050, which would represent a significant increase from 2.1 billion tons in 2023 [1,2]. Globally, more than 43% of solid waste is improperly managed through incineration, open burning, illegal dumping, and unregulated landfilling, contributing to severe environmental and public health risks [3]. Poor waste management practices and the rapid rise in the production of waste is not only leading to environmental pollution but also imposes substantial economic and health-related challenges, particularly in developing regions [4,5]. The effectiveness of waste management heavily depends on the proper classification and segregation at the source that can promote recycling, reduce landfill dependence, and achieve sustainability goals. However, traditional manual sorting approaches face several key limitations, as identified by [6]. These limitations include labor-intensive and time-consuming processes which involve humans for waste categorization. This process also includes human error and inconsistency in classification, and can lead to health risks for workers handling hazardous materials [7]. For instance, the United Nations Environment Program (UNEP) estimates that manual waste handling contributes to 15–20% of workplace injuries in the recycling sector [4]. Moreover, manual waste classification also incurs high operational costs, requires more time, lacks the scalability for handling increasing waste volumes, and highlights the urgent need for automating this process. Recent investigations show that manual waste sorting systems can sort 30 to 40 recyclable items per minute, whereas AI-powered sorting mechanisms can classify up to 160 items per minute, offering 4–5-fold improvement in real-world scenarios [8]. Moreover, the cost of manual waste management remains high due to labor, health insurance, and low scalability in handling waste volume surges [9].

**Figure 1:**
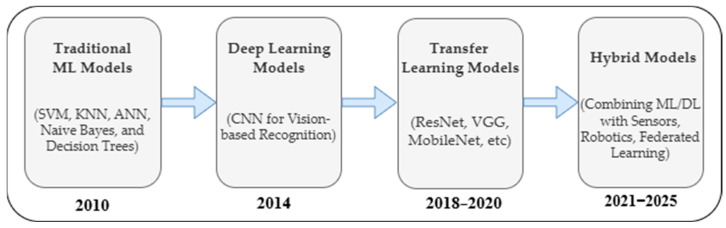
Evolution of AI techniques in waste classification.

To address these challenges, automated waste classification systems powered by AI have emerged as an alternative to manual waste classification. Over the last decade, AI-based techniques for waste classification have evolved by advancing and optimizing both machine learning and deep learning techniques. In early 2010, traditional ML models such as support vector machines (SVMs), K-nearest neighbors (KNNs), and decision trees were frequently adopted for waste classification tasks due to their simplicity and interpretability [10]. As they gained prominence, convolutional neural networks (CNNs) began to dominate by 2014, particularly in vision-based waste recognition scenarios [11]. Between 2018 and 2020, transfer learning approaches—such as ResNet, VGG, and MobileNet—enabled high-performance models that could generalize well even with limited training data [9,12]. More recently, post-2020 efforts moved toward hybrid systems, combining ML/DL models with different AI techniques as well as with sensor integration, robotic automation, and federated learning to achieve real-time and scalable classification in smart waste management applications [8,9]. Figure 1 illustrates this evolution of AI-based techniques in waste classification.

These AI-driven waste classification systems employ various machine learning (ML), deep learning (DL), and hybrid models to enhance the accuracy and efficiency of identifying different waste types. These techniques utilize vast datasets to train models that can classify waste based on visual, infrared, and multispectral data. However, the effectiveness of these AI models significantly depends on the availability and quality of publicly accessible datasets, which serve as the foundation for training and evaluating classification algorithms [13]. Despite the advancements in AI technologies, several challenges persist in achieving robust and real-time AI-based waste classification. Issues such as dataset imbalance, variations in waste appearance, domain adaptation problems, and high computational costs remain significant barriers to the widespread adoption and implementation of automated waste classification systems. Recognizing these ongoing challenges, numerous researchers have explored a variety of AI-driven approaches to enhance waste classification systems. In the literature, significant advancements in waste management are demonstrated, and numerous surveys have been conducted across the different aspects of waste classification. Table 1 illustrates existing literature reviews and survey papers, highlighting their contributions and the areas that remain insufficiently addressed.

The field of waste management and classification has evolved significantly over the past two decades, with various research approaches focusing on different aspects of the problem. For instance, the application of machine learning in municipal solid waste management was explored by [11], covering publications from 2000 to 2020 and analyzing algorithms from traditional machine learning to deep learning approaches. However, this research did not consider hybrid algorithm types, nor did it explore the use of publicly available datasets or perform comparative evaluations of algorithmic performance across different datasets. Additionally, another researcher, Ref. [12], narrowed the focus to smart waste collection systems (2016–2021), emphasizing IoT integration and real-time monitoring, but did not focus on AI-based techniques for waste collection and separation. Besides these, Ref. [15] conducted a bibliometrics-based review covering publications from 2000 to 2019, primarily focusing on traditional waste classification technologies and research trends. This study provided valuable insights into geographical contributions and journal impact, but did not address the emerging role of artificial intelligence in waste classification and management.

In recent years, a significant shift towards technological approaches is evident in recent surveys. For example, Ref. [9] conducted a comprehensive survey on deep learning applications in waste detection and classification (2000–2022), examining various architectures like YOLO, R-CNN, and ResNet. Similarly, Ref. [10] provided a systematic review of automatic separation systems (2017–2023), focusing on the integration of sensors and computing devices with machine learning models. More recently, Ref. [13] examined AI-driven service design for garbage classification (2019–2023), highlighting practical implementations like BinBin Helper and ZRR2 robot. Furthermore, building on behavioral aspects, Ref. [19] presented an extensive systematic review spanning from 1965 to 2022, analyzing individual waste separation behaviors through theoretical frameworks like theory of planned behavior (TPB) and norm activation model (NAM). This study emphasized the human factors in waste management but did not explore technological solutions for automation.

Upon examining existing review studies, it is evident that none of the systematic literature reviews (SLRs) in this research domain have provided a detailed focus on the types of waste and their classifications. Furthermore, SLRs that have discussed classifications did not discuss machine learning (ML), deep learning (DL) and hybrid methods, and provided future directions based on these classifications across different datasets. Our study addresses these gaps by covering the most recent developments (2020–2025) and presenting a comprehensive analysis of waste classification methods, including traditional machine learning techniques, advanced deep learning architectures, and novel hybrid models. In addition, we present detailed comparisons of the accuracy rates achieved across different datasets, and provide practical insights into the implementation of AI-based waste classification systems. Specifically, this study investigated the datasets used for waste classification, categorizes AI-based algorithms into three distinct types (machine learning-based, deep learning-based, and hybrid models), explores each category by analyzing the algorithms implemented by different researchers alongside the datasets applied and the accuracies achieved, and finally proposes a phased roadmap outlining short-term, mid-term, and long-term research priorities. The roadmap emphasizes improvements in accuracy, model efficiency, and the sustainable integration of AI in smart waste management systems. The remainder of this paper is organized as follows. Section 2 describes the methodology employed for the systematic literature review, including review planning, search protocol, research questions, and inclusion and exclusion criteria. Section 3 presents the results and analysis, covering publicly available waste classification datasets and AI-based techniques, categorized into machine learning, deep learning, and hybrid models. Section 4 discusses future research directions, emphasizing challenges such as dataset scarcity, model efficiency, explainable AI, and federated learning. Finally, Section 5 concludes the study by summarizing key findings and contributions.

## 2. Methodology

This study follows Kitchenham guidelines [21] for conducting systematic reviews and follows the PRISMA (Preferred Reporting Items for Systematic Reviews and Meta-Analyses) guidelines [22] for reviewing and selecting articles. The Kitchenham model structures the review process into three sequential phases: planning, conducting, and reporting. The planning phase involves identifying the need for a review, formulating research questions, and developing a detailed search protocol. The conducting phase implements the search strategy across major databases such as Web of Science, IEEE Xplore, Scopus, ScienceDirect, and Google Scholar, followed by rigorous filtering based on relevance, quality, and reproducibility. Finally, the reporting phase focuses on synthesizing and presenting the key findings with clarity and traceability.

**Figure 2:**
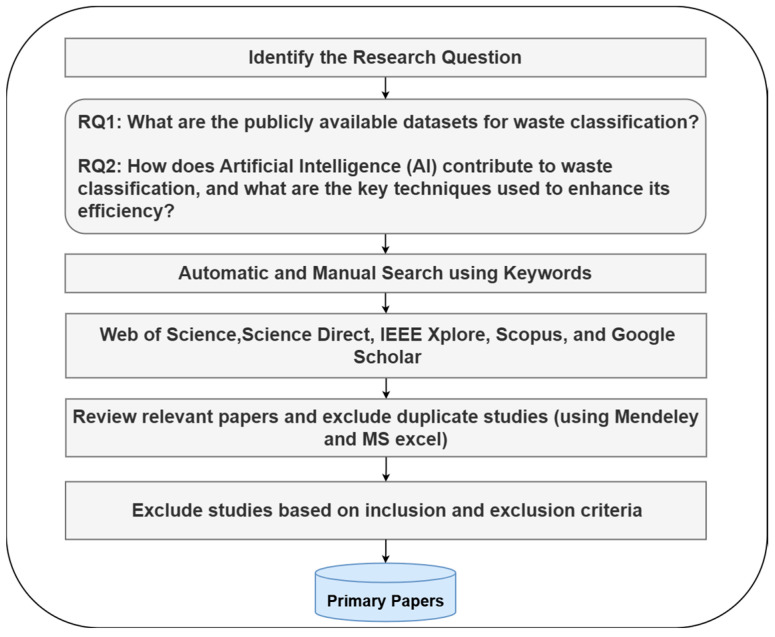
Review protocol.

**Figure 3:**
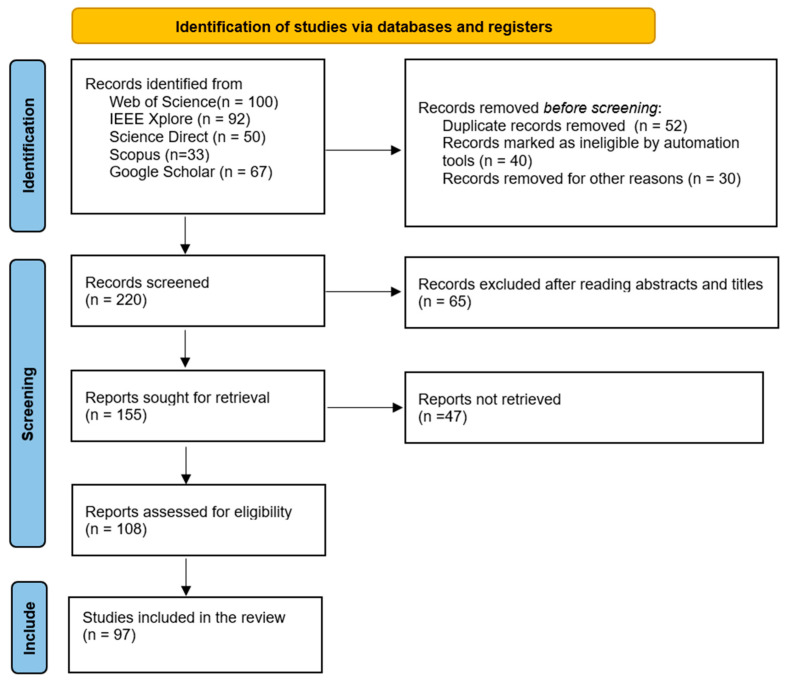
Flow diagram adapted from PRISMA [22].

Complementing this, the PRISMA protocol enhances methodological transparency by guiding the processes of identification, screening, and inclusion through well-defined eligibility criteria and structured flow diagrams. PRISMA consists of four core stages: defining eligibility criteria, selecting articles using Boolean queries, extracting and assessing data quality, and synthesizing the final dataset. A total of 385 studies were initially retrieved, with 97 meeting the inclusion criteria. The overall review protocol followed in this study is summarized in Figure 2 whereas, the PRISMA selection flow is illustrated in Figure 3. The inclusion criteria focused on peer-reviewed studies (2020–2025) specifically addressing AI-based waste classification, while non-English, theoretical, and outdated studies were excluded.

### 2.1. Planning the Review

The planning phase of this SLR follows the Kitchenham guidelines [21], ensuring a structured and transparent review process. It involves developing a systematic search protocol (Section 2.1.1) and formulating research questions with defined objectives (Section 2.1.2). These steps ensure a methodical and well-documented approach to analyzing AI-based waste classification.

#### 2.1.1. Search Protocol

To ensure a comprehensive and structured literature review, this study follows the Preferred Reporting Items for Systematic Reviews and Meta-Analyses (PRISMA) guidelines [22]. The search protocol is designed to systematically identify, screen, and select relevant studies related to waste classification using AI techniques. This methodology ensures transparency and reproducibility in selecting high-quality research articles. Figure 1 shows the review protocol which has been followed in this research for the primary papers’ selection.

##### Data Sources, Search Strategy, and Data Extraction

The primary data sources for this review included well-known academic databases such as Web of Science (for multidisciplinary research publications), IEEE Xplore (for AI and computer science-related research), Scopus (for indexing peer-reviewed articles), ScienceDirect (for access to engineering and environmental science literature), and Google Scholar (for broad academic literature). Besides these, Kaggle and GitHub were also explored for publicly available waste classification datasets.

According to PRISMA guidelines, the first step in the systematic review process is identification. This involves determining the relevant keywords to perform searches in the databases mentioned above. The study employed a two-step search strategy, namely a primary search and a secondary search. In the primary search, automated keyword-based searches were conducted in each database using structured Boolean queries optimized for comprehensive retrieval. Keywords included phrases such as “Machine learning in waste classification”, “Deep learning in waste classification”, “Hybrid AI models for waste classification”, “Public datasets for waste classification”, “Smart waste management”, and “Sustainability in waste management.” Boolean search strings such as (“Waste Classification” OR “Waste Sorting” OR “Garbage Recognition”) AND (“Machine Learning” OR “Deep Learning” OR “Artificial Intelligence”) AND (“Dataset” OR “Benchmark” OR “Accuracy Comparison”) were used across Web of Science, IEEE Xplore, Scopus, and Google Scholar. The comprehensiveness and representativeness of keywords were ensured through a three-step strategy aligned with Kitchenham and PRISMA guidelines. First, a preliminary review of highly cited papers from the period 2020–2025 was conducted to extract frequently used technical terms and domain-specific terminology related to AI-driven waste classification (e.g., “CNN”, “YOLO”, “smart bins”, “garbage detection”). Second, multiple synonym groups and variations (e.g., “garbage” vs. “waste”, “sorting” vs. “classification”) were tested across the selected databases to examine result variations. Queries yielding both redundant and unique papers were shortlisted to improve recall and precision. Third, test queries were piloted on smaller subsets within IEEE and ScienceDirect to evaluate hit rates and scope coverage, ensuring that no dominant taxonomy or classification technique was excluded. These measures ensured that the search strings adequately reflected the landscape of literature relevant to machine learning, deep learning, and hybrid models in waste classification. In terms of quality control, dual-researcher validation was applied to all search outputs and inclusion decisions. The Boolean strings were revised iteratively based on retrieval feedback, and screening was conducted independently by two researchers before merging and resolving discrepancies through consensus with a third. This rigorous, multi-pass review and inclusion control ensured that the methodology is both transparent and scientifically systematic, as recommended by Kitchenham and PRISMA frameworks.

In the secondary search, additional refinement was carried out to ensure comprehensive literature coverage. This included manual reviews of the reference lists from initially selected papers to identify additional relevant studies, as well as backward and forward citation analysis. To further expand the search scope, keywords such as “AI-based waste classification”, “Smart waste bins”, and “IoT in waste classification” were also incorporated. The outcomes of both search strategies were systematically managed and documented using the Mendeley reference management tool (version 2.132), which facilitated the automated tracking, organization, and citation of all sources. Each retrieved article underwent a thorough screening and evaluation process. Initially, duplicate entries across databases were identified and removed. This was followed by title and abstract screening, where studies not directly related to AI techniques, waste classification methods, or relevant datasets were excluded from further analysis.

For the eligible studies, a standardized data extraction form was developed to ensure consistency in capturing key information across sources. The extracted data points included: paper title, publisher name, year of publication, type of algorithm applied (machine learning, deep learning, or hybrid), specific technique or model used, dataset name, dataset composition and breakdown, reported model accuracy, and a concise description of the methodology and key findings. This structured extraction was essential for performing a detailed comparative evaluation of how various AI techniques have been utilized to enhance waste classification outcomes.

The review protocol, illustrated in Figure 2, details the step-by-step process followed in this research for the selection of primary papers, beginning with the identification of research questions through to the final inclusion of primary studies. By incorporating a strategic approach to data sourcing, the careful planning of the search process, and meticulous data extraction, this methodology section ensures that the systematic literature review is built on a robust foundation. This comprehensive approach aims to provide an extensive analysis of the current landscape and contribute significant insights into the application of machine learning in enhancing waste management practices, consistent with the PRISMA guidelines [22].

To provide a visual summary of the article selection process, a PRISMA flowchart (Figure 3) was developed. This illustrates the number of records identified, screened, excluded, and finalized for inclusion in this study. Finally, the methodological quality of each included paper was assessed based on the clarity of its approach, the completeness of its technical reporting, and the relevance and suitability of its AI methodology in the context of waste classification.

#### 2.1.2. Research Questions

The research questions (RQs) for this study were formulated based on the gaps identified in previous literature and the objectives of this systematic review. The purpose of these questions is to provide a structured analysis of AI-based waste classification methodologies, datasets, and performance metrics.

The key research questions addressed in this study are:

**RQ1:**  *What are the publicly available datasets for waste classification?*

This question aims to identify and analyze the datasets used in AI-driven waste classification research, covering their characteristics, sources, and applicability.

**RQ2:**  *How does artificial intelligence (AI) contribute to waste classification, and what are the key techniques used to enhance its efficiency?*

This question explores the various AI techniques (machine learning, deep learning, and hybrid models) utilized for waste classification, their effectiveness, and their impact on improving accuracy and automation in waste management systems. These research questions serve as the foundation for the systematic literature review, guiding the selection, categorization, and evaluation of relevant studies.

### 2.2. Inclusion and Exclusion Criteria

In this study, the specific criteria have been established for the precise inclusion and exclusion of papers from several databases as described in Figure 3. These criteria ensue most relevant and high-quality studies that allow the review to comprehensively cover recent advancements while maintaining scientific rigor. Table 2 summarizes these criteria:

Following the above-mentioned criteria, several examples of excluded papers are provided. For instance, Ref. [23] was excluded for being published in Chinese, failing the language criterion. Ref. [24] was excluded despite being a relevant study since it was an academic report rather than a peer-reviewed study. Older studies such as [25] were excluded based on the publication date. Lastly, Ref. [4] was excluded for offering general discussions unrelated to AI or machine learning. These examples demonstrate the strict adherence to the defined criteria in Table 2 and reinforce the methodological rigor of the selection process.

Following the review protocol and inclusion and exclusion criteria, our study considered those studies published between 2020 and 2025 and used some dataset for waste classification and discussed the accuracy achieved by specific algorithm. Studies with insufficient dataset documentation or incomplete methodological descriptions were interpreted more cautiously when synthesizing our findings. Two researchers (Farnaz Fotovvatikhah and Muhammad Umair) separately screened the titles and abstracts of the studies based on a set of inclusion criteria, and any disagreement between the researchers was solved by discussion with the second and third researcher (Ismail Ahmedy and Rafidah Md Noor). Such selection procedures reduce the biases of selection as these involve several researchers. Moreover, Figure 3 illustrates the PRISMA flow diagram that illustrates the detailed process followed in this study for the selection of primary papers, from the identification of initial articles through the screening and eligibility assessment, to the final inclusion of studies.

**Figure 4:**
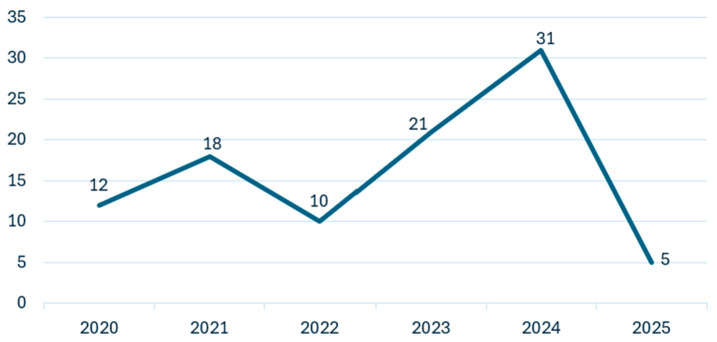
Number of published papers on waste classification by year (2020–2025).

Figure 3 illustrates the systematic selection process following the PRISMA guidelines. In this study, a total of 342 records were identified from various databases including Google Scholar (n = 67), Web of Science (n = 100), Science Direct (n = 50), and IEEE Xplore (n = 125). During the identification phase, 122 records were removed: duplicate records (n = 52), records marked as ineligible by automation tools (n = 40), and records removed for other reasons (n = 30). This resulted in 220 records being screened. After reviewing abstracts and titles, 65 records were excluded, leaving 155 reports sought for retrieval. Of these, 47 were not retrieved, and the remaining 108 reports were assessed for eligibility. Ultimately, 97 studies met all criteria and were included in the review. After applying the inclusion and exclusion criteria, a total of 97 publications were selected for this systematic literature review. Figure 4 illustrates the distribution of these publications across the study period (2020–2025).

The analysis reveals a significant upward trend in research activity related to AI-based waste classification, with publications increasing from 12 papers in 2020 to 31 papers in 2024. This growing trend reflects the increasing interest in applying machine learning and deep learning techniques to waste classification challenges. The notable peak in 2024 (31 publications) represents a 47.6% increase from 2023 (21 publications), indicating accelerated research momentum in this field. The limited number of publications in 2025 (5 papers) is attributed to the review being conducted early in the year. This temporal distribution highlights the rapidly evolving nature of AI applications in waste management systems and the growing recognition of their potential to address critical environmental challenges.

**Figure 5:**
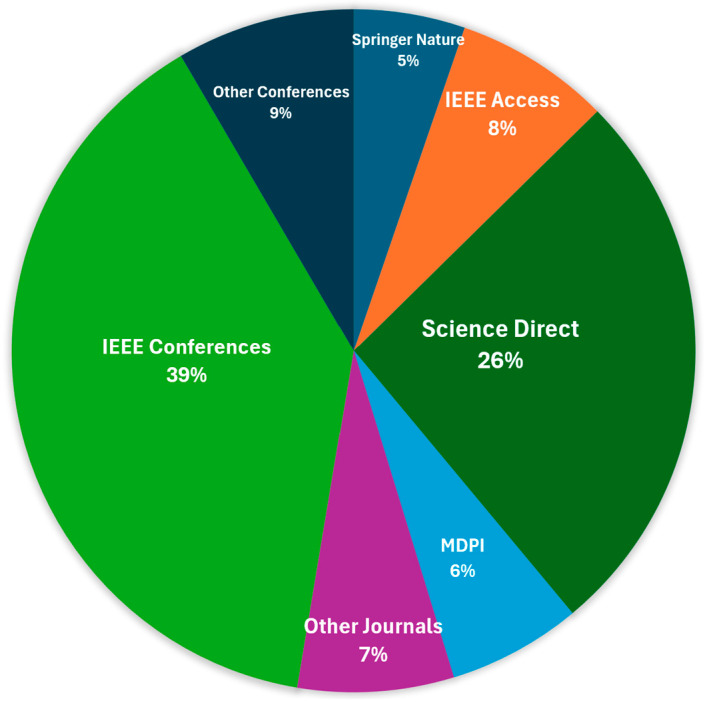
Distribution of selected papers by publication source.

Furthermore, Figure 5 illustrates the distribution of selected papers across different publishers and venues. The analysis reveals that a significant portion of the research on AI-based waste classification has been published in IEEE venues, with IEEE Conferences accounting for 39% of all publications, followed by IEEE Access at 8%, totaling 47% of all included studies. Science Direct represents the second largest source with 26% of publications, indicating substantial interest from interdisciplinary environmental and technological journals. The remaining publications are distributed across various sources, including Other Conferences (9%), Other Journals (7%), MDPI (6%), and Springer Nature (5%).

## 3. Results and Analysis

This section presents a comprehensive analysis of the reviewed studies across four major dimensions relevant to AI-based waste classification. Section 3.1 explores publicly available waste datasets, highlighting their structure, diversity, and commonly reported limitations in the literature. Section 3.2 systematically reviews machine learning and deep learning techniques applied for waste classification, focusing on the types of algorithms used, associated datasets, and reported accuracy levels. Section 3.3 investigates the hybrid approaches that integrate multiple AI methods to improve the classification performance and address the limitations of individual techniques. Section 3.4 extends the analysis by examining real-world implementations and assessing the technology readiness level (TRL) of selected systems, showcasing how several AI models have progressed from conceptual designs to operational prototypes. This structure facilitates a logical, comparative overview of how various AI-driven methods are being implemented, optimized, and transitioned towards practical deployment in the domain of waste classification.

### 3.1. Publicly Available Waste Dataset for Waste Classification

In the field of waste management and waste classification, many researchers have employed various methods to collect and classify waste data into different categories. To build effective AI models for waste classification, high-quality datasets are essential, as they serve as the foundation for training, testing, and evaluating AI-based models. These datasets enable researchers to develop benchmark algorithms tailored to specific waste characteristics. This subsection provides an overview of publicly available datasets that have been widely used in the literature, highlighting their structure, diversity, and labeling schemes, as well as the limitations commonly reported by researchers. These datasets vary in size, composition, and the types of waste images they contain, which are crucial for developing robust machine learning and deep learning models. Table 3 outlines key details of these datasets, including information about the number of images they contain and the types of waste categories featured, along with their respective references. Understanding these datasets is critical for selecting appropriate data sources and benchmarking classification accuracy across different studies.

Based on the dataset analysis presented in Table 3, a comprehensive comparative analysis has been conducted to examine the dataset size, coverage, and applicability of datasets in AI-based waste classification research.

#### 3.1.1. Dataset Size and Coverage

Based on the analysis, the dataset has been divided into three categories: large-scale datasets, medium-scale datasets, and small-scale datasets. The large-scale datasets, containing more than 30,000 images, represent the most comprehensive collections in the waste images collection. The Open Litter Map Dataset stands as the largest, containing over 100,000 real-world litter images with geotagging information, making it particularly valuable for training models that need to perform well across diverse environmental conditions [32]. The WasteRL Dataset contains 57,000 images and provides extensive coverage of organic waste, recyclables, and hazardous materials, complemented by detailed bounding box annotations that enhance its utility for object detection and segmentation tasks [27]. The TriCascade WasteImage Dataset, comprising 35,264 images, offers specialized coverage across nine distinct categories, including industrial, medical, and hazardous waste, making it particularly suitable for specialized industrial applications [40]. In the medium-scale category, several datasets containing between 10,000 and 30,000 images offer balanced coverage of waste types. The GIGO Dataset (25,000 images) [35] and Kaggle Waste Classification Dataset (22,500 images) [28] focus on common household and commercial waste, with the latter specifically designed for binary classification between organic and recyclable materials. The NWNU-TRASH Dataset (18,911 images) [12] and Baidu Garbage Classification Dataset (17,690 images) [38] provide a comprehensive coverage of recyclable materials, with the Baidu dataset notably featuring 158 detailed sub-categories and multilingual labeling. The VN Trash Dataset, containing over 13,000 images, adds a valuable regional perspective by focusing on Southeast Asian waste types and packaging materials.

The small-scale datasets, while more limited in size, often offer specialized focus or high-quality annotations that make them valuable for specific applications. The ZeroWaste Dataset (4661 images) [36] and WaDaBa Dataset (4000 images) [31] exemplify this trend, with the former providing high-quality standardized images of common recyclable materials and the latter specializing in plastic waste classification under controlled conditions. The CompostNet Dataset (2751 images) [26] focuses specifically on organic and compostable waste, while the TrashNet Dataset (2400 images) [24], despite its smaller size, remains widely cited as a benchmark dataset in waste classification research due to its early standardization of waste imagery.

Across all these datasets, there is a notable trend towards increasing specialization and annotation quality. The larger datasets typically offer broader coverage and environmental diversity, which makes them suitable for developing robust, general-purpose classification models. Medium-scale datasets often balance comprehensive coverage with specific focus areas, while smaller datasets tend to excel in specialized applications or serve as well-documented benchmarks. This diversity in dataset characteristics provides researchers with multiple options for training and evaluating waste classification models, although considerations of dataset size, quality, and specificity must be carefully weighed against specific research requirements and applications.

#### 3.1.2. Platforms for Dataset Collection, Annotation, and AI Model Deployment

Following the overview of waste image datasets in Section 3.1.1, this section explores the platforms and tools that have been adopted for collecting data, annotating images, training models, and deploying intelligent waste classification systems. Across the reviewed studies, dataset acquisition tools such as Kaggle, Google Images, Roboflow, and TrashBox were widely used. Kaggle datasets were commonly utilized due to their structured format and class diversity [43,44,45,46,47,48,49,50,51]. Researchers also extracted additional samples from Google Images to enhance dataset realism [40,52,53], and Roboflow served as both a source and annotation platform compatible with YOLO and TensorFlow pipelines [40,42] TrashBox, an open access repository on IEEE Dataport, was also integrated in several studies focused on object-level classification [34,41].

Annotation practices varied considerably across the literature. Some studies relied on manual folder-based labeling structures, especially for binary or multi-class classification tasks [24,36,44,47,54,55,56]. For pixel-wise segmentation, LabelMe was employed to create detailed masks for semantic regions, notably in DWSD [42] and in other segmentation-focused research such as [57,58,59]. Object detection-based research often employed custom XML-based labeling systems to define bounding boxes and classes [49,60].

Table 4 summarizes the training platforms reported in the reviewed literature, listing only those studies in which the authors explicitly mentioned the simulation tools or development environments employed. TensorFlow and PyTorch emerged as the most dominant frameworks, appearing across a wide spectrum of deep learning-based waste classification implementations. Additionally, other platforms such as MATLAB, Keras, and Scikit-learn were also utilized, particularly in studies involving traditional machine learning algorithms or hybrid model configurations.

**Figure 6:**
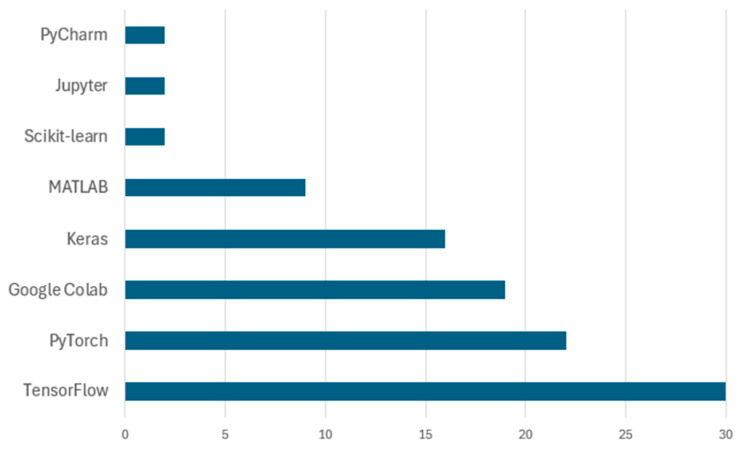
Frequency of training platform usage in reviewed studies.

The frequency of the use of each platform is visualized in Figure 6, which reflects the popularity of different tools across all reviewed papers. TensorFlow appears as the leading platform with 30 documented uses, followed by PyTorch and Google Colab. This distribution aligns with the growing accessibility of deep learning frameworks and GPU-powered environments. Google Colab, in particular, supported scalable model training for researchers without high-end local infrastructure. While MATLAB and Scikit-learn were less frequently used, they provided robust environments for traditional learning models and algorithm prototyping.

Although the majority of works were developed in simulated settings, some studies progressed towards embedded deployment. Frameworks such as TensorFlow Lite, Jetson Nano, and Raspberry Pi enabled the real-time deployment of lightweight CNNs like MobileNet and YOLOv7-tiny [49,61,62]. These systems demonstrated promising performance in indoor waste sorting and intelligent bin applications. However, large-scale deployment in uncontrolled environments remains underexplored and serves as a key area for future research. While these platforms and tools have greatly accelerated the development of AI-driven waste classification systems, they also expose several recurring challenges related to data quality, class imbalance, reproducibility, and annotation consistency—issues that are further analyzed in the following section on dataset limitations and challenges.

#### 3.1.3. Dataset Limitations and Challenges

Despite the availability of various waste classification datasets, several significant limitations and challenges persist that impact their effectiveness in real-world applications. The analysis of these datasets reveals four primary areas of concern: data imbalance and representation, environmental variability, standardization issues, and scalability challenges.

Data imbalance represents a significant challenge across most waste classification datasets. While larger datasets like the Open Litter Map Dataset (>100,000 images) and WasteRL Dataset (57,000 images) offer substantial data volumes, they often exhibit uneven distribution across waste categories. Common waste types such as plastic and paper typically dominate these datasets, while hazardous materials and specialized waste categories remain underrepresented. For instance, the TrashNet Dataset, despite its widespread use, shows significant imbalance with certain categories, containing fewer than 400 images and potentially leading to biased model performance. This issue was also been identified in datasets such as WaDaBa and TrashBox, where imbalanced class distributions contribute to poor generalization for minority classes [79,85,109]. These limitations directly influence how AI models perform in practical settings. For example, datasets like TrashNet and WaDaBa, with their highly imbalanced classes, often produce misleadingly high accuracy for dominant waste types such as plastic or paper, while misclassifying minority categories like glass, textile, or hazardous waste. Studies such as [86,93,112] have shown that architectures like ResNet-50 or MobileNetV2 tend to overfit on frequent classes in such datasets, resulting in biased classification outcomes and poor generalization. Similarly, models trained on datasets with consistent lighting and backgrounds may perform well in closed settings but fail in outdoor or cluttered environments. This is particularly evident when comparing model performance across structured datasets like TrashNet and more diverse, real-world collections like Open Litter Map or TACO. Annotation quality also introduces a variability that includes poorly labeled or inconsistently annotated images, as seen in some crowdsourced datasets, degrading precision and recall.

Furthermore, applying the same AI model across different datasets often yields varying performance results due to dataset-specific factors. For instance, DenseNet-121 achieved 96.4% accuracy when trained on VNTrash and TrashNet [86], but only 85.5% on the WaDaBa dataset [86], which is more imbalanced and less diverse under lighting conditions. Likewise, MobileNetV2 consistently scored above 93% on structured datasets like Kaggle [69] and TrashNet [112], but dropped to 87.3% on WaDaBa [86], highlighting the sensitivity of lightweight models to dataset quality. These discrepancies reveal that model accuracy is heavily influenced not just by architecture, but by dataset characteristics like class distribution, real-world diversity, and labeling consistency. Thus, evaluating models across multiple datasets is essential to understand their generalizability and real-world applicability. These examples underline the need for robust training strategies such as class rebalancing, domain adaptation, and synthetic data augmentation that can mitigate the impact of dataset limitations on model performance.

Environmental variability and real-world conditions pose another crucial challenge. Most controlled datasets, such as TrashNet and the WaDaBa Dataset, capture images under consistent lighting and controlled conditions, which may not reflect real-world scenarios. While the Open Litter Map Dataset includes diverse environmental conditions through its crowdsourced approach, it introduces inconsistencies in image quality and annotation reliability. The TACO dataset attempts to address this by including images of waste in natural environments, but with only 1500 images, it may not capture the full spectrum of environmental variations. Studies such as [59,93] have highlighted the need for datasets collected under heterogeneous environmental conditions to ensure that models can adapt to real-world deployment settings. Standardization issues across datasets present significant obstacles for comparative research and model development. Different datasets employ varying categorization schemes, annotation methods, and image quality standards. For example, the Baidu Garbage Classification Dataset uses 158 sub-categories [38], while simpler datasets like the Kaggle Waste Classification Dataset [28] employ binary classification. This lack of standardization makes it challenging to combine datasets or compare model performance across different studies. Additionally, inconsistencies in image resolution, formatting, and metadata structure further complicate the integration of multiple datasets for comprehensive model training. Several studies have emphasized the need for unified dataset formats and cross-compatible taxonomies to support model transferability [79,109].

The scalability and maintenance of datasets emerge as ongoing challenges in waste classification research. The dynamic nature of waste production and evolving material types necessitate regular dataset updates and expansions. However, most existing datasets represent static collections, potentially becoming outdated as new packaging materials and waste types emerge. The TriCascade WasteImage Dataset attempts to address this by including modern waste categories such as e-waste and medical waste [40], but maintaining comprehensive, up-to-date datasets requires significant resources and coordination. Furthermore, the cost and effort required for detailed annotations, particularly for datasets with bounding boxes and segmentation masks like the WasteRL Dataset, pose challenges for dataset expansion and updates.

In response to these limitations, several recent studies and future research directions have proposed alternative strategies such as dataset fusion, synthetic image generation, few-shot learning, and self-supervised techniques to reduce the reliance on large manually annotated datasets [92,93,109]. Moreover, federated learning and domain adaptation are gaining traction as viable methods to enable cross-dataset generalization without requiring unified data collection protocols. Overall, these limitations underscore the critical need for more inclusive, well-annotated, and scalable datasets to ensure the robustness and practical applicability of AI-based waste classification models. Tackling these challenges not only enhances the accuracy and generalizability of classification algorithms but also lays the groundwork for the AI-driven modeling techniques discussed in the next section on AI-based techniques for waste classification.

### 3.2. AI-Based Techniques for Waste Classification

**Figure 7:**
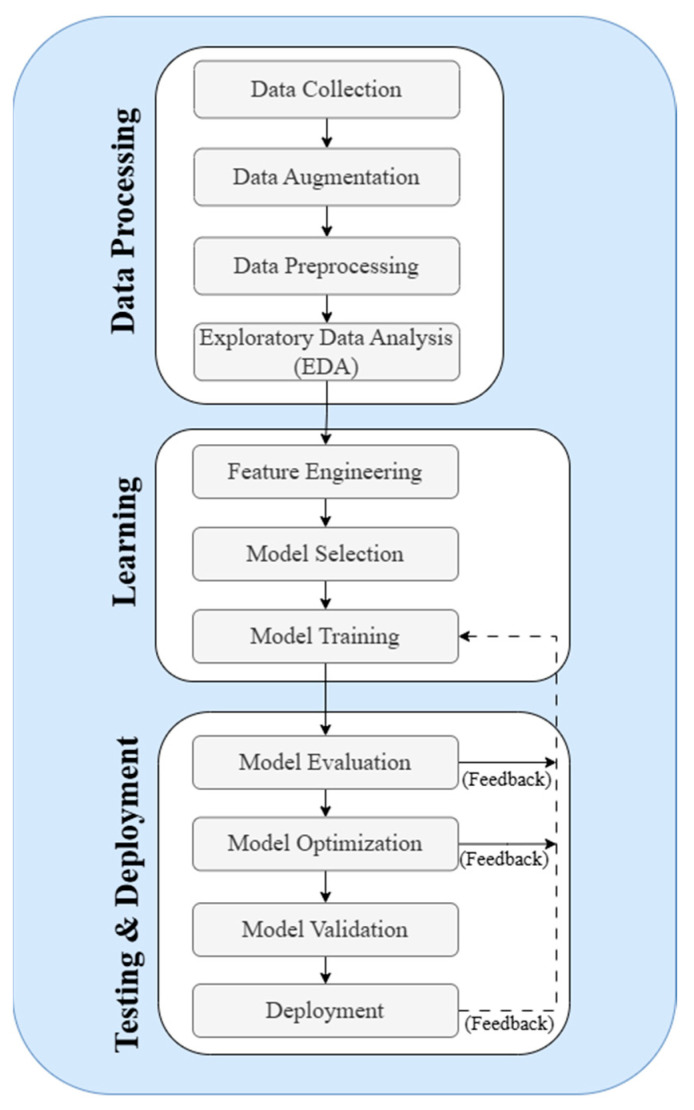
Machine learning pipeline for waste classification.

AI-based techniques for waste classification are commonly categorized into three major groups: machine learning (ML), deep learning (DL), and hybrid models. Machine learning approaches are typically based on manual feature engineering, where specific characteristics such as texture, shape, or color are extracted and then used by algorithms like support vector machines (SVMs), decision trees, or k-nearest neighbors (k-NN) for classification. These methods are often lightweight and interpretable, making them effective for structured datasets. In contrast, deep learning approaches utilize architectures like convolutional neural networks (CNNs) to automatically learn hierarchical features from raw image data, thereby achieving higher accuracy for image-based classification tasks, especially on large-scale or unstructured datasets. Hybrid models combine the strengths of both ML and DL using CNNs for feature extraction and feeding the results into other models such as SVM, random forest, or even sequence-based models like Bi-LSTM or transformer-based networks. These approaches aim to improve performance, robustness, and generalizability in real-world waste classification systems. The following subsection presents a comprehensive review of the AI-based techniques adopted in waste classification research, based on 97 primary studies. These techniques are broadly categorized into machine learning, deep learning, and hybrid approaches, each presenting distinct advantages depending on the data characteristics and classification goals. This section explores how these models have been implemented, which datasets were used, what performance metrics were reported, and how each technique contributes to improving waste identification accuracy. The analysis includes both conventional algorithms and modern deep architectures, with comparisons drawn from published benchmarks and methodological trends. The classification of waste serves as a crucial indicator in establishing effective waste management strategies, such as recycling and resource recovery. Typically, waste classification involves manual sorting at the point of waste generation, such as at waste transfer stations or material recovery facilities. This process is labor-intensive, time-consuming, and prone to errors, particularly when workers handle hazardous substances, which can pose significant health risks [6]. A viable solution to these challenges is the adoption of AI-based techniques, which involve different kinds of algorithms that utilize the visual and physical features of collected materials to train systems to perform automatic waste stream classification. Figure 7 illustrates an enhanced machine learning pipeline for waste classification, which is structured into three key phases: data processing, learning and testing, and deployment. In the data processing phase, the process begins with data collection of waste, where images of various waste types are gathered. Data augmentation techniques, such as rotation and scaling, are applied to increase the diversity of the dataset, thereby improving the model’s ability to generalize. The data are then pre-processed to clean and standardize it, ensuring consistency and suitability for analysis. Following this, exploratory data analysis (EDA) is performed to uncover patterns and insights within the data, which guide the subsequent steps. During the learning phase, feature engineering is conducted to create new features or modify existing ones that better capture the characteristics of the waste data. Feature selection then identifies the most relevant features to improve model accuracy and efficiency. The process continues with model selection, multiple AI-based classification models—such as CNNs, transfer learning architectures, and ensemble-based classifiers—are evaluated and compared [16]. The selected model is then trained on the prepared dataset. Finally, in the testing and deployment phase, the trained model undergoes an evaluation to assess its performance using metrics such as accuracy, precision, and recall. Importantly, performance feedback loops have been integrated into the pipeline, as illustrated in Figure 6. These loops enable iterative refinement, where insights from model evaluation and validation are routed back to retrain and optimize the model, ensuring higher classification robustness. This feedback-based optimization loop is considered essential in modern smart waste classification frameworks [113]. Once the model was validated, it was deployed to perform real-time waste classification, thereby automating and streamlining the waste management process.

**Figure 8:**
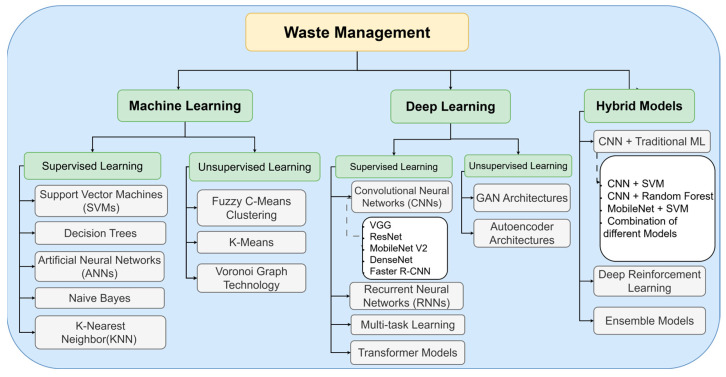
Waste classification analysis using AI-based techniques.

The AI techniques used in waste classification primarily fall into three major categories: machine learning-based approaches, deep learning-based approaches, and hybrid AI models, as shown in Figure 8.

The taxonomy shown in Figure 8 illustrates the three primary approaches to waste classification using AI. Machine learning-based approaches include traditional supervised learning methods like support vector machines (SVMs), decision trees, artificial neural networks (ANNs), and naive Bayes, alongside unsupervised techniques such as fuzzy C-means clustering and K-means. These methods have established track records in classification tasks with structured data.

Deep learning-based approaches represent more advanced architectures, centered around convolutional neural networks (CNNs) and their variants (VGG, ResNet, MobileNet, DenseNet). This category also includes specialized architectures like recurrent neural networks (RNNs) for sequential data processing, multi-task learning frameworks for handling multiple classification objectives simultaneously, and transformer models for complex feature extraction. The unsupervised branch in deep learning primarily focuses on GAN architectures and autoencoder systems, which excel at learning data representations without explicit labels.

**Figure 9:**
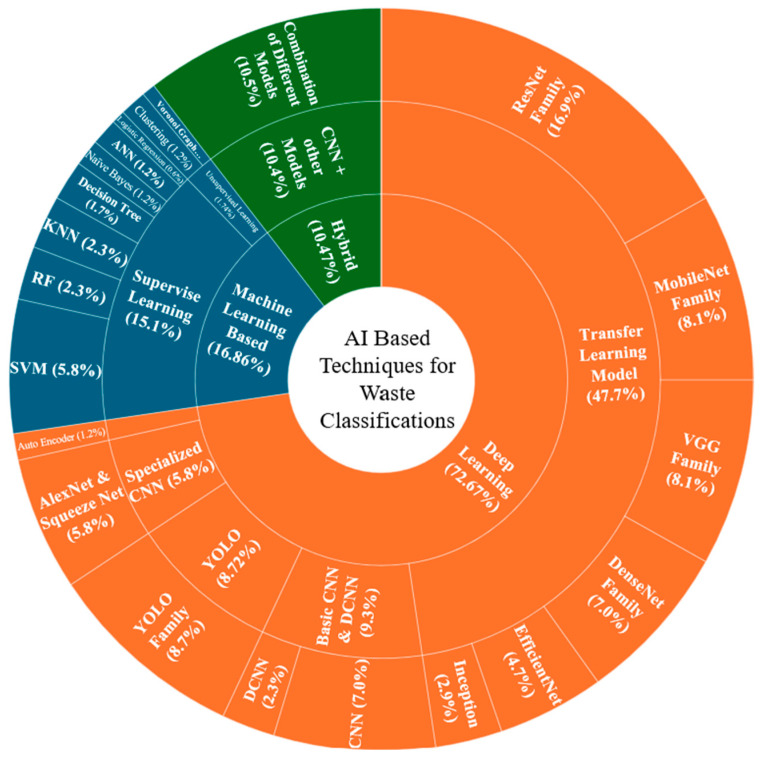
Distribution of AI-based techniques for waste classification.

Hybrid models combine the strengths of multiple approaches, particularly the integration of CNNs with traditional machine learning algorithms (ANN, SVM, random forest) or with optimization techniques. This category also includes ensemble models and deep reinforcement learning applications, which have shown promise in adaptive waste classification systems. These hybrid approaches often achieve superior performance by leveraging both the feature extraction capabilities of deep learning and the robust classification abilities of traditional machine learning algorithms. Following the presentation of Figure 7, which illustrates the taxonomy of AI techniques in waste classification, Figure 9 provides a comprehensive quantitative analysis of the 97 reviewed papers’ methodological distribution. The circular visualization reveals several significant patterns in the adoption of AI techniques for waste classification.

In the deep learning domain, transfer learning approaches dominate the research landscape, with ResNet Family being the most widely implemented architecture (29 papers), followed by YOLO Family (15 papers) for object detection. MobileNet Family and VGG Family each appear in 14 studies, while DenseNet Family and basic CNN architectures are represented in 12 papers each. EfficientNet and Inception architectures are less frequently utilized, appearing in eight and five papers, respectively. Within the machine learning-based approaches, supervised learning techniques show greater adoption compared to unsupervised methods. SVM emerges as the most popular traditional machine learning algorithm, implemented in 10 studies. KNN and RF each appear in four papers, while decision trees and ANN are used in three and two papers, respectively. Unsupervised learning approaches, including clustering algorithms and Voronoi graph theory, are less frequently employed, appearing in only three papers collectively. Hybrid models, combining CNN with traditional machine learning approaches, demonstrate significant adoption with 16 papers implementing various combinations of different models. This substantial representation of hybrid approaches suggests a growing recognition of the benefits of combining multiple AI techniques for enhanced waste classification performance.

#### 3.2.1. Machine Learning-Based Approaches

Machine Learning (ML) techniques have been widely applied in waste classification due to their ability to identify patterns in structured datasets. Machine learning (ML) can be classified into two types, supervised learning and unsupervised learning.

Supervised learning is a widely used machine learning approach in which several types of ML models are trained on specific labelled datasets. This technique utilizes historical data (can be of any type, i.e., paper, Bottle, cardboard, metal, or plastic) where the outcomes are known, and trains the models to predict the classification of new data accordingly. This process allows the model to learn the mapping between input features and output labels. The learning process begins with data collection and labelling the data, where each instance is tagged with the correct waste category (such as plastic, metal, or glass). Relevant features, such as color, texture, and shape, are then extracted from the data to serve as inputs to the learning algorithm. After data extraction, various supervised learning algorithms are employed (including support vector machines (SVMs), decision trees, artificial neural networks (ANNs), random forest (RF), and K-nearest neighbors (KNNs)) to associate specific feature patterns with the correct waste categories. After model training, it is evaluated on a separate validation dataset to assess the accuracy, using metrics such as accuracy, precision, and F1-score. Once trained, the model can predict the waste category for new, unseen data by applying the learned function to the input features. This process automates the waste classification, leading to a reduction in the need for manual sorting and enhancing classification accuracy.

**Figure 10:**
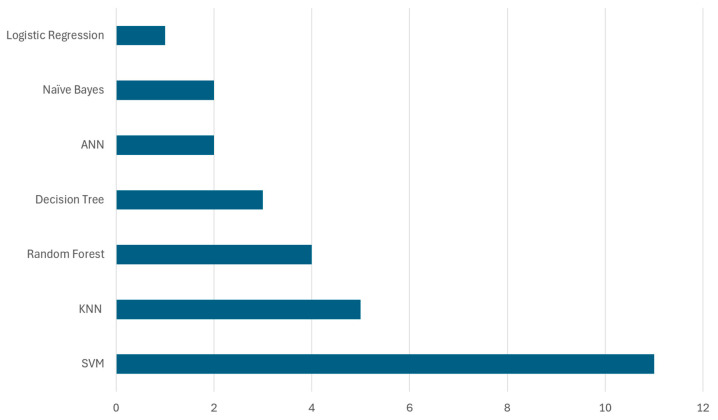
Frequency of machine learning-based algorithms for waste classification.

Figure 10 illustrates the frequency of various supervised learning models used by the researchers in waste classification. For instance, SVMs have been extensively applied in waste classification, specifically being used in eleven papers, showing a significant focus, whereas the KNN algorithm was used in five and random forest was used in four papers, and decision tree, ANN, naïve Bayes, and logistic regression were not used that much and only used in less than four papers. This visualization helps quantify the research interest and focus on specific supervised learning models in the context of waste classification.

Table 5 provides a detailed overview of supervised learning techniques for waste classification. This table illustrates the types of algorithms used, information about the datasets used on which these algorithms were applied, and the performance metrics achieved.

As illustrated in Table 5, and inside Figure 10, SVM was widely utilized in several studies for waste classification, achieving accuracy levels ranging from 62.5% to 94.8%. Notably, Ref. [97] applied SVM to the TrashNet dataset, achieving 85% accuracy. This study compared multiple algorithms, including random forest, decision tree, and CNN (ResNet34), and utilized image preprocessing techniques such as grayscale conversion and resizing. In contrast, Ref. [114] experimented with SIFT and PCA feature extraction, demonstrating that PCA did not significantly enhance the classification performance, achieving 65% accuracy. The highest recorded accuracy for SVM was 94.8%, obtained in [43] by integrating CNN-based feature extraction, indicating the benefits of hybrid approaches.

KNN was applied in multiple studies, with a reported accuracy ranging from 77% to 94.1%. In [110], KNN was used with IoT-based waste monitoring systems, achieving 77% accuracy, while [44] implemented KNN in a hybrid CNN-ANN model, leading to 94.1% accuracy. This suggests that KNN performs significantly better in image-based datasets when combined with deep learning approaches. Similarly, random forest demonstrated accuracy between 55% and 95.2%, depending on the dataset characteristics. The lowest accuracy (55%) was reported in [97] when applied to the TrashNet dataset. In contrast, Ref. [110] integrated random forest with IoT-based sensor data, achieving 85.29% accuracy, and Ref. [44] achieved 95.2% accuracy using a hybrid deep learning approach. These findings emphasize that random forest benefits from feature engineering and integration with CNN-based models.

Decision tree models exhibited accuracy ranging from 65% to 88.32%. In [97], decision tree achieved 65% accuracy, whereas Ref. [115] applied it to construction site waste classification, attaining 88.32% accuracy. This variation suggests that decision tree models are more effective when applied to structured datasets rather than complex image classification tasks. Beside these, ANN models showed high classification performance, with [64] reporting 98% accuracy on municipal waste data, and Ref. [57] achieving 96.1% accuracy on waste characterization data. Meanwhile, naïve Bayes had mixed performance. Ref. [110] applied naïve Bayes to IoT-based waste classification, achieving 84.1% accuracy, while Ref. [44] recorded only 51.2% accuracy on the Kaggle dataset, indicating that naïve Bayes is better suited for structured sensor data rather than image-based classification. Logistic regression was employed in IoT-based waste classification, achieving 80% accuracy in [110]. The results suggest that logistic regression is reliable for structured classification tasks but less effective for high-dimensional waste classification problems.

Unsupervised learning is a type of machine learning where models are trained on data without labelled outcomes. In the context of waste management, this approach is particularly useful for discovering hidden patterns or intrinsic structures within the data that have not been pre-categorized. Techniques such as clustering are commonly used to group the data based on similarities and differences, and are commonly used in unsupervised learning. For instance, unsupervised learning can be used to identify different types of waste materials based on their physical properties or to optimize waste collection routes without prior knowledge of specific categories. Despite its potential, unsupervised learning has not received as much attention in waste classification as other approaches like supervised learning, deep learning, or hybrid models have received attention. Still, there is a space of research in unsupervised learning, particularly in scenarios where labeled data are scarce or where the goal is to explore data-driven insights.

**Figure 11:**
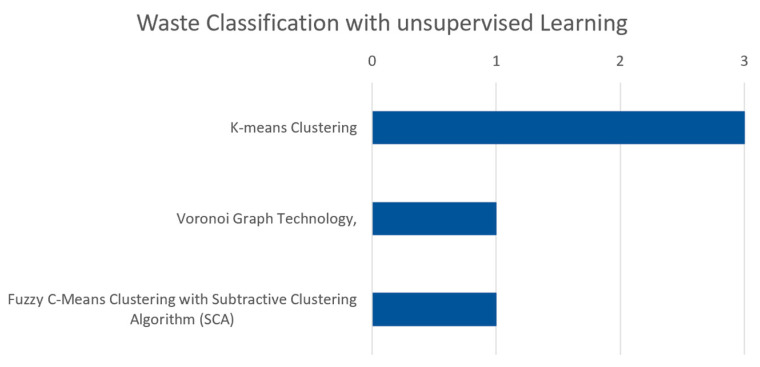
Frequency of unsupervised learning models in waste classification research.

Figure 11 illustrates the frequency of various unsupervised learning models discussed in this section. For example, K-means clustering is discussed in three papers, indicating a notable emphasis on this technique within the field of waste classification. In contrast, Voronoi graph technology is covered in two papers, and fuzzy C-means clustering with subtractive clustering algorithm (SCA) is mentioned in one paper. Moreover, Table 6 illustrates the application of unsupervised learning techniques in waste management, including the models used, the datasets they were applied to, and the key findings from each study.

Unsupervised learning has been applied in waste classification to explore patterns and classifications without the need for labeled data. K-means clustering was frequently utilized across various studies. For instance, Ref. [54] used K-means to analyze a multi-objective solid waste dataset, effectively identifying patterns in the data. Similarly, Ref. [116] applied this technique to GIS data for evaluating potential landfill sites, demonstrating its effectiveness in multi-criteria decision analysis. Another study [111] introduced a new approach called HOM CS, using K-means to classify fuel samples based on physical properties, optimizing fuel usage for energy conversion. Additionally, Voronoi graph technology, combined with clustering algorithms, was employed by [106] to optimize the urban garbage collection networks in Beijing, significantly improving efficiency from 74.9% to 95.6%. This study highlights the potential of unsupervised learning in optimizing complex systems. Another advanced method, fuzzy C-means clustering with the subtractive clustering algorithm (SCA), was applied by [117] to classify oily waste from marine oil spill operations, improving waste management strategies. These studies showcase the versatility of unsupervised learning techniques in handling complex and high-dimensional data in waste management, though challenges like ensuring data accuracy and adapting algorithms to various contexts remain prevalent.

#### 3.2.2. Deep Learning-Based Approaches

Deep learning is an advanced extension of supervised learning that has revolutionized artificial intelligence by enabling machines to learn complex patterns directly from large datasets. Traditional machine learning algorithms require manual feature extraction, which is time-consuming and not that perfect, whereas deep learning models automatically discover features from raw data, making them particularly effective for tasks such as classification and detection. Deep learning models utilize deep neural networks, which are composed of multiple layers of interconnected neurons. These multiple layers consist of an input layer, hidden layers, and an output layer. The input layer receives and processes raw data, such as images of waste items, with each neuron corresponding to specific features within the received dataset of images. Hidden layers transform these features into increasingly complex representations, which allow the model to capture subtle distinctions between different types of waste. The output layer then classifies the waste into categories such as cardboard, glass, metal, organic, paper, plastic, and trash. During training, the model fine-tunes the connections between neurons through backpropagation, minimizing classification errors and improving its ability to accurately sort waste into these specific categories.

**Figure 12:**
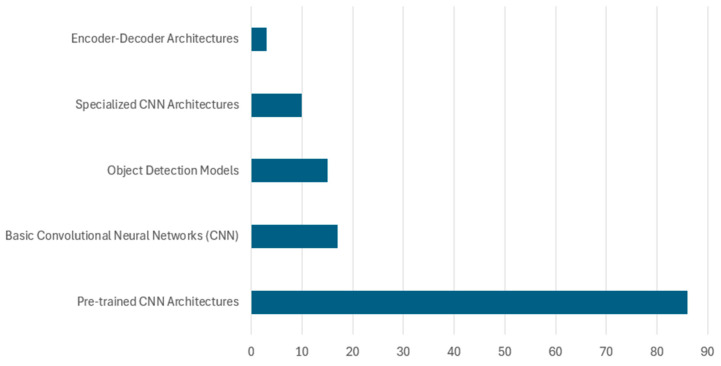
Frequency of deep learning models in waste classification research.

This study reveals various deep learning approaches applied to waste classification, which can be categorized into five main architectural types as shown in Figure 12. Among these five architectural types, the pre-trained CNN architectures dominate with 86 implementations, highlighting researchers’ strong preference. These pre-trained models include various iterations in VGG, ResNet, DenseNet, MobileNet, and EfficientNet families, which have proven particularly effective for waste classification tasks due to their robust feature extraction capabilities and transfer learning advantages. This popularity is largely due to the availability of these pre-trained networks, which are easy to fine-tune and retrain on domain-specific datasets, making them highly suitable for small and medium-scale waste classification applications. Basic convolutional neural networks (CNNs) represent the second most common approach, with 17 implementations. These include custom-designed architectures specifically created for waste classification. Object detection models rank as the third most used approach, counting for 15 implementations, primarily comprising YOLO variants and faster R-CNN. These models are particularly valuable in real-time waste detection and localization scenarios, where identifying and localizing multiple waste items simultaneously is crucial. Furthermore, specialized CNN architectures, including modified versions of AlexNet and SqueezeNet, comprise 10 implementations. These architectures have been specifically adapted to address the unique challenges of waste classification, often incorporating modifications to improve efficiency or accuracy for specific waste types. The least represented category is encoder–decoder architectures with three implementations, suggesting an emerging area for future research in waste classification. While currently underutilized, these architectures show potential for detailed waste segmentation and feature extraction tasks.

In the following sections, each of these architectural types will be explored in detail.

##### Basic CNN and DCNN Models

**Figure 13:**
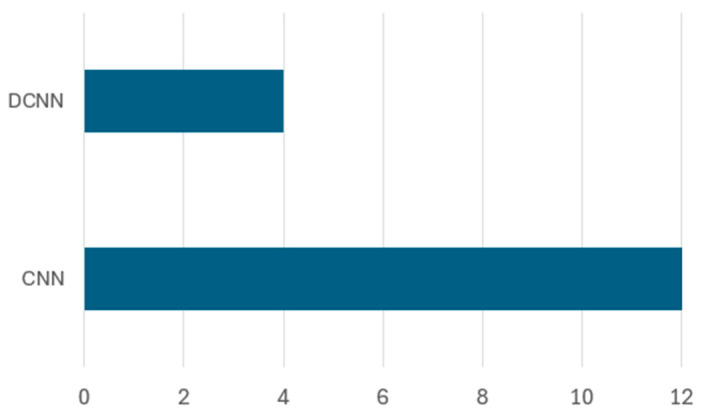
Frequency of basic CNN and DCNN models.

CNNs and DCNNs are widely applied in waste classification due to their ability to automatically extract hierarchical features from images. CNN models consist of convolutional layers that capture spatial patterns, pooling layers that reduce dimensionality, and fully connected layers that classify the waste categories. DCNN models extend the traditional CNN architecture by incorporating additional layers, which improve feature extraction and enhance performance for complex datasets. These models are particularly effective for sorting different waste types such as plastic, metal, paper, and organic waste. Their ability to generalize across diverse datasets makes them ideal for real-world waste classification applications. Figure 13 illustrates the distribution of basic CNN and DCNN architectures implemented in waste classification studies.

The analysis reveals that CNN architectures were predominantly used, with 12 studies implementing CNN models compared to 4 studies utilizing DCNN approaches. Table 7 presents various research contributions utilizing CNN and DCNN architectures for waste classification. The table highlights the used model, the dataset name, the dataset breakdown, and the classification accuracy by different studies.

As illustrated in Table 6, most of the CNN and DCNN models achieved more than 90% accuracy. For instance, Ref. [118] implemented a CNN model on a dataset containing 15,000 images across twelve categories, achieving an accuracy of 95.4%. This was primarily due to the integration of feature extraction layers with extensive data augmentation techniques that improved generalization across multiple waste categories. Similarly, Ref. [45] employed additional augmentation techniques such as contrast stretching, image rotation, and noise addition, achieving an accuracy of 94.4%. These findings suggest that data augmentation plays a crucial role in improving CNN performance, particularly when dealing with imbalanced datasets. Despite these accuracies, CNN models are highly dependent on model architecture and hyperparameter tuning. The study by [24] reported significantly lower accuracy (22%) due to the suboptimal hyperparameter settings and limited data augmentation. This highlights the importance of fine-tuning CNN architectures and preprocessing strategies to prevent performance degradation. Furthermore, CNN models have been compared with traditional machine learning approaches such as SVM, random forest, and decision trees. The study by [43] found that SVM outperformed CNN (94.8% vs. 83%), suggesting that CNNs may struggle when trained on limited datasets due to their reliance on large-scale labeled data. However, a hybrid CNN-based model integrating VGG16 with FastNet-34 outperformed both standalone CNN and SVM models, achieving 95.6% accuracy. This suggests that hybrid models, which combine deep learning with traditional machine learning classifiers, can significantly enhance waste classification performance.

In another comparative study, Ref. [97] implemented a CNN alongside SVM, random forest, and decision tree models, and found that CNN achieved 90% accuracy. This further reinforces the effectiveness of CNNs in image-based classification tasks. The performance of deep learning models can also be improved by integrating them with advanced training techniques and transfer learning. Deep CNN (DCNN) models have demonstrated notable improvements over standard CNNs, particularly in handling complex waste classification tasks. The study by [66] introduced the ConvoWaste model with Capsule-Net, achieving 98% accuracy in real-time waste segregation. Capsule networks enhanced the model’s ability to detect spatial relationships between waste objects, making it particularly useful in cluttered environments where multiple waste items overlap. Similarly, Ref. [119] utilized transfer learning, comparing DCNN performance with pre-trained models such as VGG16, VGG19, MobileNetV2, DenseNet121, and EfficientNetB0. The study reported an accuracy of 93%, demonstrating that leveraging pre-trained architectures significantly improves classification performance, particularly when working with smaller datasets. Furthermore, in another study, Ref. [58] utilized CNNs and R-CNNs to detect and classify e-waste, achieving an accuracy between 90% and 97%. Moreover, dataset diversity has also played a crucial role in CNN performance. Models trained on larger, more diverse datasets consistently reported higher accuracy. For example, Ref. [47] trained a CNN model on the Kaggle garbage classification dataset, achieving an accuracy of 96%, reinforcing that extensive labeled datasets enhance classification performance. On the other hand, studies trained on smaller, less diverse datasets often encountered generalization challenges. The study by [55] attempted to overcome this limitation by augmenting the TACO dataset with additional custom images, achieving an accuracy of 92%. This suggests that dataset expansion through augmentation can enhance model robustness and improve classification accuracy.

A growing trend in waste classification research is the integration of CNNs with machine learning classifiers such as SVM, random forest, and K-nearest neighbors. Hybrid models have consistently outperformed standalone CNNs, as evidenced by studies such as [120], which developed JONET, a hybrid AI model integrating CNN, SVM, and random forest. This hybrid approach achieved 92.7% accuracy, demonstrating that combining deep learning and traditional machine learning techniques enhances waste classification performance. Similarly, Ref. [50] refined CNN architectures with additional Conv2D layers, obtaining an accuracy of 97.58%, showing that refining convolutional layers can further boost classification accuracy.

##### Pre-Trained CNN Architectures (Transfer Learning)—VGG Family

Pre-trained CNNs have become a dominant approach in waste classification due to their ability to leverage transfer learning, significantly improving their classification accuracy without the need to train models from scratch on large datasets. Among these, the VGG family, particularly VGG-16 and VGG-19, has been widely adopted due to its deep architecture and strong feature extraction capabilities. VGG models, composed of sequential convolutional layers followed by max pooling and dense layers, are especially effective in capturing spatial hierarchies in waste images.

**Figure 14:**
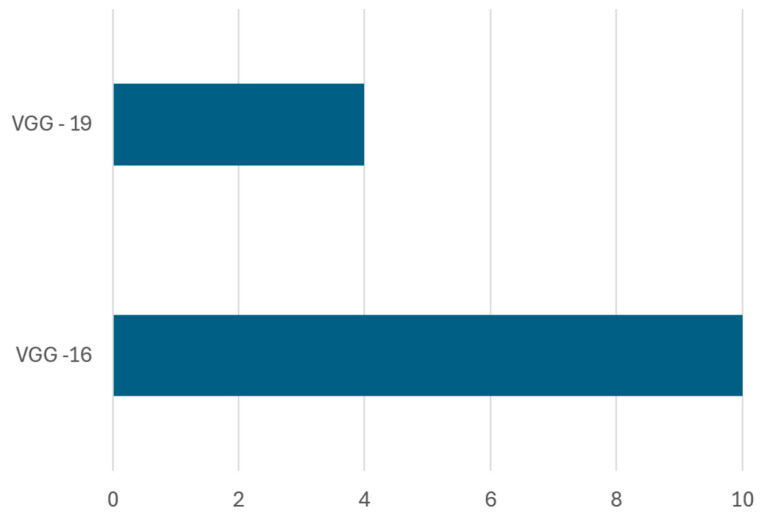
Frequency of VGG family for waste classification.

Frequency and dataset coverage: Across reviewed studies, VGG-16 has emerged as the most frequently used architecture, appearing in 10 out of 14 implementations involving the VGG family. In contrast, VGG-19 has been used less often, appearing in only four studies. Figure 14 highlights the frequency of each architecture in existing research, and Table 8 provides detailed information on dataset types, dataset splits, achieved accuracy, and references.

Performance on Benchmark Datasets: The performance of VGG-16 has been notably strong across various datasets. For instance, Ref. [49] reported 98% accuracy on the Kaggle dataset for organic and recyclable waste, attributing the improvement to data augmentation techniques and the use of the Adam optimizer with binary cross-entropy loss. Similarly, Ref. [61] achieved 98.15% accuracy by implementing VGG-16 in a real-time embedded system using Raspberry Pi and additional sensors, demonstrating the potential for low-cost, IoT-integrated smart waste solutions with high TRL (technology readiness level), likely at TRL 6–7, based on prototype validation in real-world environments.

Several studies also examined hybrid approaches involving VGG-16. For example, Ref. [52] combined VGG-16 with YOLO for real-time detection and achieved 93% accuracy using techniques like shifting, padding, and cropping for data augmentation. Likewise, Ref. [51] fine-tuned VGG-16 alongside ResNet50v2, obtaining 93.49% accuracy, showing the benefits of architectural diversity. In [46], a comparative study with ResNet50 and Inception-V3 revealed that VGG-16 achieved the highest accuracy of 88.42% on the OrgalidWaste dataset.

Evaluation of VGG-19 performance: Although VGG-16 generally outperforms VGG-19, studies show that deeper architectures can be beneficial under specific conditions, like [48] utilized VGG-19 on the Kaggle garbage classification dataset, achieving 91% accuracy, whereas Ref. [45] reported only 56% accuracy using the same model, indicating that VGG-19 requires careful hyperparameter tuning and large-scale datasets to perform optimally. Interestingly, Ref. [108] employed deep feature fusion techniques, integrating VGG-19 with ResNet101, GoogleNet, and AlexNet, obtaining 85.17% accuracy, which suggests that combining multiple deep models can improve classification performance through feature fusion.

Transfer Learning Using VGG Architectures: The effectiveness of transfer learning is further reinforced in studies where VGG architecture has been compared against other deep learning models. For instance, Ref. [43] compared VGG-16 with SVM, CNN, and a hybrid CNN-based model using FastNet-34, showing that the hybrid model achieved 95.6% accuracy, surpassing the standalone VGG-16 and SVM models. Similarly, Ref. [82] combined VGG-16 with Bi-LSTM and Bayesian hyperparameter tuning, achieving 87.25% accuracy on the TrashNet dataset, demonstrating the potential of hybrid deep learning architectures for enhancing waste classification performance. Similarly, Ref. [83] examined the impact of transfer learning on VGGNet-16 and ResNet-50 for organic and residual waste classification, reporting 95.6% accuracy on a custom dataset. In contrast, studies such as [50] trained a custom CNN and compared it against pre-trained deep learning models (VGG-16, ResNet50, MobileNetV2, InceptionV3, and EfficientNetB0), achieving 87.57% accuracy, highlighting that dataset augmentation plays a crucial role in improving classification outcomes.

These results confirm that VGG-16 remains one of the most reliable baseline models in AI-based waste classification. It performs exceptionally well when enhanced through transfer learning, hybridization, and embedded system integration. Its broad adoption, high accuracy, and successful deployment in pilot implementations suggest that VGG-16-based models are approaching mid-to-high TRL levels, making them viable for scalable real-world applications.

##### ResNet Family: Transfer Learning Using Pre-Trained CNN Architectures

**Figure 15:**
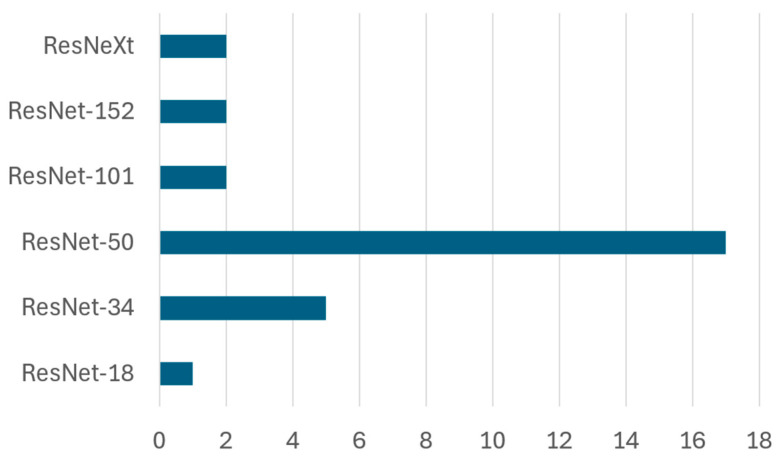
Frequency of ResNet family for waste classification.

ResNet (Residual Network) architectures are widely adopted in waste classification due to their ability to mitigate vanishing gradient issues, thereby supporting the training of deep models capable of learning complex hierarchical features. The ResNet family encompasses models such as ResNet-18, ResNet-34, ResNet-50, ResNet-101, ResNet-152, and ResNeXt, each varying in depth and computational complexity. Several studies have applied these architectures for classification and object detection tasks, leveraging data augmentation, hybrid models, and deep feature fusion techniques to enhance accuracy. Figure 15 illustrates the implementation frequency of these architectures in the reviewed literature.

Implementation Trends and Dataset Coverage: Among the ResNet family, ResNet-50 dominates the landscape, appearing in 16 studies, followed by ResNet-34 in six studies. Other variants such as ResNet-101, ResNet-152, and ResNeXt are used less frequently, with 1–2 implementations each. These architectures have been applied across datasets like TrashNet, Kaggle garbage, WaDaBa, ScrapNet, OrgalidWaste, and various custom datasets, as summarized in Table 9, highlighting dataset details, dataset breakdown, and classification accuracy.

Performance of Shallow ResNet Variants (ResNet-18 and ResNet-34): ResNet-18 and Resnet-34 are the lightweight variants that are commonly used for their efficiency and fast training times which make them suitable for real-time and embedded applications. ResNet-18 was applied by [56], and achieved an accuracy of 95.87% on the TrashNet dataset by incorporating a self-monitoring module for dynamic parameter tuning, showing promise in adaptive waste classification. ResNet-34 was frequently applied in [67,82,84], reporting accuracy levels above 91%. In particular, Ref. [84] achieved 94.64% accuracy by introducing dropout layers, batch normalization, and a cyclic learning rate, while [67] obtained 96.27% accuracy on combined VNTrash and TrashNet datasets, emphasizing the benefits of multi-source data training. Additionally, Ref. [53] proposed a customized ResNet-34 model with multi-feature fusion and a novel activation function, achieving an exceptional 99.96% accuracy, indicating that structural modifications significantly enhance the performance.

Dominance and Versatility of ResNet-50: ResNet-50 offers an optimal balance between depth and computational complexity, making it a preferred architecture for high-accuracy waste classification across varied datasets. Among all variants inside the ResNet family, ResNet-50 remains the most commonly applied model due to its balance between depth and computational efficiency. Several studies, including [48,68,82,109], implemented ResNet-50, reporting accuracy levels ranging from 85.54% to 96.83%. In particular, Ref. [109] classified plastic waste using multiple CNN models, finding that ResNeXt slightly outperformed ResNet-50 (87.44% vs. 85.54%), emphasizing that deeper architectures may be better suited for certain waste classification tasks. Similarly, Ref. [87] combined ResNet-50 with YOLO for real-time object detection, achieving 96.83% accuracy for classification and 61% mAP for multi-object detection, reinforcing that ResNet-50 excels in single-object classification but requires additional architectures for multi-object detection. Furthermore, Ref. [83] achieved an accuracy of 96.6% by fine-tuning the model on a custom dataset for organic and residual waste classification. In contrast, Refs. [45,46] reported 66.67% and 50.28% accuracy, respectively, indicating that data augmentation techniques such as contrast stretching and image rotation may not always lead to substantial accuracy improvements. Refs. [50,88] evaluated ResNet-50 alongside VGG16, MobileNetV2, InceptionV3, and EfficientNetB0, finding that ResNet-50 achieved 94.34% accuracy, highlighting its generalization across varied data types. These findings suggest that ResNet-50-based models are reaching their mid-to-high TRLs (technology readiness levels 6–7), making them suitable for real-world pilot deployments in smart waste classification systems.

Deeper Variants—ResNet-101 and ResNet-152: These deeper models are intended to extract more intricate features, but their benefits are often constrained by the need for extensive training data and higher computational cost. These models are used in studies such as [69,108], achieving 87.76% and 70.7% accuracy, respectively. These findings suggest that while deeper models capture more complex representations, they may require significantly larger datasets and computational resources to outperform shallower ResNet architectures. Similarly, Ref. [88] applied ResNet-152 on the ScrapNet dataset, reporting 79.11% accuracy, further highlighting the computational challenges associated with deeper networks.

Emerging Alternatives—ResNeXt: ResNeXt enhances feature reuse and representation by introducing grouped convolutions, potentially improving accuracy without significantly increasing complexity. ResNeXt was applied in [85,86] on the WaDaBa dataset, achieving 87.44% accuracy. Ref. [121] also showed more accuracy than ResNet-50 in the same study. This suggests that ResNeXt may offer performance advantages over traditional ResNet models for specific waste classification tasks.

The ResNet family, particularly ResNet-50, is widely adopted in waste classification due to its strong performance, ease of transfer learning, and balance of computational complexity. Studies show that, while shallower networks like ResNet-18 and 34 perform well with proper tuning, custom ResNet-34 architectures with feature fusion can outperform deeper models. Additionally, ResNeXt represents a promising direction, warranting further investigation. As a result, ResNet-based models—especially when paired with hybrid or object detection systems, are well positioned for real-world waste classification deployments.

##### Pre-Trained CNN Architectures (Transfer Learning)—DenseNet Family

DenseNet (densely connected convolutional networks) is a deep learning architecture designed to enhance feature reuse and mitigate vanishing gradient problems by introducing direct connections between all layers. Unlike conventional CNNs, where each layer feeds only into the next, DenseNet connects each layer to every other layer in a feed-forward fashion, improving information flow and model efficiency. These densely connected pathways enable more effective gradient propagation, which is particularly advantageous in deep networks for tasks such as waste classification.

**Figure 16:**
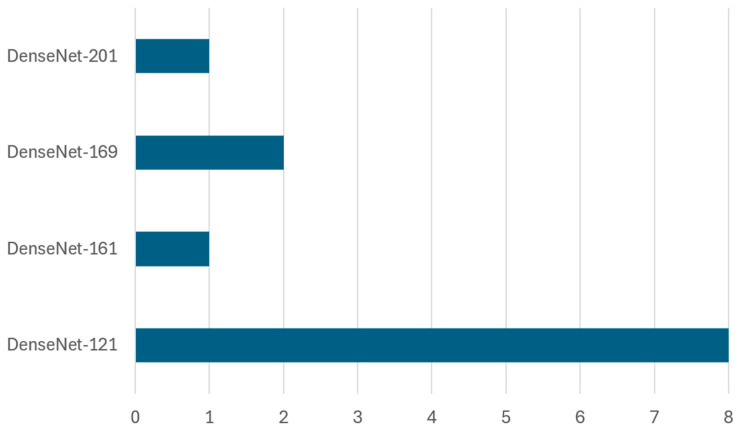
Frequency of DenseNet family for waste classification.

Implementation Trends and Dataset Use: As shown in Figure 16, DenseNet-121 is the most frequently adopted variant, appearing in eight studies, followed by DenseNet-169 in two studies. DenseNet-161 and DenseNet-201 were each applied in one study. These models have been evaluated on various datasets including TrashNet, WaDaBa, Kaggle, VNTrash, and several custom datasets. Table 10 summarizes these implementations along with dataset usage, split ratios, classification accuracy, and corresponding references.

Effectiveness of DenseNet-121: DenseNet-121, a lighter model with high efficiency and deep connectivity, has been widely used for its ability to generalize well with relatively fewer parameters. Several studies [67,68,70,71] demonstrated classification accuracies ranging from 91% to 96.43% using this model. For instance, Ref. [68] applied DenseNet-121 on a custom dataset and reported 91% accuracy, while Ref. [67] fine-tuned the model using transfer learning, achieving 93.30% on the TrashNet dataset. The results highlight that transfer learning significantly enhances the model’s ability to generalize across diverse waste categories. In [84], the model’s performance was enhanced through data augmentation and genetic algorithm (GA)-based optimization, increasing the accuracy to 94.02%. That study also employed Grad-CAM visualization for interpretability, reinforcing the importance of explainable AI techniques. Similarly, Ref. [70] applied DenseNet-121 on TrashNet, and achieved 94.44% accuracy through dropout layers, batch normalization, and cyclic learning rate optimization, while Ref. [71] used an extended dataset (VNTrash and TrashNet), and obtained 96.43% accuracy, suggesting that dataset diversity significantly contributes to the classification performance. In plastic waste classification, Refs. [85,121] tested DenseNet on the WaDaBa dataset, each reporting 85.5%+ accuracy. Although ResNeXt slightly outperformed DenseNet in these cases, DenseNet remained competitive due to its parameter efficiency and strong feature representation.

Performance of Deeper Variants—DenseNet-161 and DenseNet-169: DenseNet-161 is a deeper and more computationally intensive model that is used when richer feature hierarchies are required. DenseNet-161 achieved the highest accuracy (97.45%) in [102], where a hybrid learning approach combining deep learning with metaheuristic algorithms for feature selection optimization was applied. This underscores the effectiveness of combining deep learning with feature engineering techniques to improve the classification accuracy. DenseNet-169, which offers a balance between depth and performance, was used in [12,84], achieving 95.66% accuracy on TrashNet and 82.80% accuracy on NWNU-TRASH, respectively. The lower accuracy in [12] was attributed to dataset inconsistencies, as NWNU-TRASH was generated through web scraping and manual photography, which introduced quality variations in the dataset.

DenseNet vs. Other CNNs and Hybrid Integration: DenseNet-121’s capabilities were further validated in [82], where it was compared with AlexNet and SqueezeNet for solid waste classification, achieving 94.15% accuracy—outperforming both models. This confirms its superior spatial feature learning ability. In addition, Ref. [120] proposed JONET, a hybrid AI model combining DenseNet-201, SVM, and random forest (RF), achieving 96.06% accuracy on TrashNet and local datasets. This suggests that hybrid architectures incorporating CNN-based feature extraction with ML classifiers improve the classification performance, particularly for structured datasets. Overall, the DenseNet architecture, especially DenseNet-121, exhibits strong classification performance in waste classification. Their success is often amplified when combined with transfer learning, data augmentation, optimization techniques, and hybrid classifiers. Their parameter efficiency and robust feature extraction make them suitable candidates for deployment in real-world intelligent waste management systems.

##### Pre-Trained CNN Architectures (Transfer Learning)—MobileNet Family

**Figure 17:**
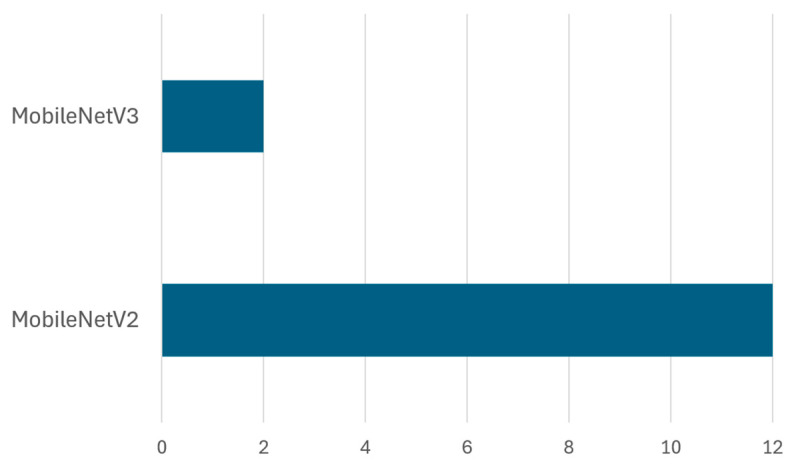
Frequency of MobileNet family for waste classification.

MobileNet is a lightweight deep learning architecture specifically optimized for mobile and edge deployment, using depth-wise separable convolutions to reduce parameters while maintaining competitive performance. Its efficiency and low computational footprint make it a strong candidate for real-time waste classification tasks, especially in resource-constrained environments [62]. The MobileNet family, including MobileNetV2, MobileNetV3, and their variations (e.g., SSD MobileNetV2 Quantized, GNet), has been extensively utilized in waste classification due to its efficiency in handling large datasets with limited computational resources. Figure 17 illustrates the implementation frequency of MobileNet variants in waste classification research.

Implementation Trends and Dataset Coverage: As shown in Figure 17, MobileNetV2 dominates the MobileNet family, appearing in 11 studies, while MobileNetV3 has been implemented in 2 studies. These architectures have been tested across datasets such as TrashNet, Kaggle, WaDaBa, VNTrash, and several custom and region-specific datasets. A detailed overview of dataset splits, usage, and accuracy is presented in Table 11.

Performance of MobileNetV2: MobileNetV2, the most frequently implemented variant in this family, is widely adopted due to its excellent trade-off between accuracy and computational efficiency, making it ideal for real-time classification tasks. Studies [48,50,62,67,68,70,72,73,74,85,121,122] have demonstrated its effectiveness, achieving classification accuracy ranging from 80% to 99.7% across different datasets. Ref. [48] applied MobileNetV2 on the Kaggle garbage classification dataset and achieved 93% accuracy, demonstrating its capability in large-scale classification tasks. Additionally, Ref. [50] compared MobileNetV2 with other pre-trained models, including VGG16, ResNet50, and EfficientNetB0, reporting 96.99% accuracy, highlighting the effectiveness of MobileNet’s feature extraction capabilities in waste categorization. A significant performance enhancement was observed in [67,70], where transfer learning techniques were applied using pre-trained MobileNetV2 models. Ref. [45] utilized MobileNetV2 on an extended dataset (VNTrash, TrashNet), achieving 96.27% accuracy, indicating that dataset expansion plays a crucial role in improving model generalization. Similarly, Ref. [70] leveraged MobileNetV2 for classification tasks, obtaining 93% accuracy on TrashNet, reinforcing that transfer learning enhances MobileNet’s classification capabilities across diverse datasets.

MobileNetV2 has also been evaluated in plastic waste classification. Refs. [85,121] applied MobileNetV2 on the WaDaBa dataset, achieving 87.35% accuracy, while ResNeXt slightly outperformed MobileNetV2 in the same study (87.44%), suggesting that MobileNetV2 performs well but may not be the optimal choice for specialized waste categories like plastic waste. Moreover, MobileNetV2 has further been integrated into real-time and IoT-based waste classification systems. Ref. [62] proposed a CNN-based smart waste management system using SSD MobileNetV2 Quantized, achieving 80% accuracy in real-time object detection on embedded devices with TensorFlow Lite. The integration of LoRa-GPS for bin monitoring in this study suggests that MobileNetV2 is highly adaptable to IoT-based waste tracking solutions. Similarly, Ref. [73] optimized MobileNetV2 with attention mechanisms and PCA for real-time waste detection on Huawei Cloud datasets, achieving 90.70% accuracy, demonstrating MobileNet’s effectiveness in real-world waste sorting systems. Beside these, Ref. [122] utilized MobileNetV2 on a custom bag classification dataset, achieving an overall accuracy of 98%, showcasing MobileNet’s efficiency in domain-specific waste classification. Another important study, Ref. [74], used MobileNetV2 for digestible and indigestible waste classification in Bangladesh, reporting the highest accuracy (99.70%) for digestible waste, reinforcing MobileNetV2’s high efficiency when combined with properly preprocessed datasets.

Performance of MobileNetV3 and Its Variants: MobileNetV3, an enhanced version focusing on even lower latency and energy consumption, has been tested less frequently but shows good results in embedded environments. For instance, Ref. [69] applied MobileNetV3 on the TrashBox dataset, achieving 85.91% accuracy, indicating that while MobileNetV3 is computationally efficient, it slightly lags behind MobileNetV2 in large-scale classification tasks. However, Ref. [75] introduced GNet, a MobileNetV3-based model, and improved its performance for garbage classification in an embedded Linux environment, achieving 92.62% accuracy, suggesting that MobileNetV3 variants can be effectively adapted for edge computing applications.

The MobileNet family, particularly MobileNetV2, demonstrates strong adaptability and competitive accuracy, especially when integrated with transfer learning and lightweight optimizations. Its effectiveness across diverse waste classification tasks, coupled with suitability for edge devices and IoT environments, positions MobileNet-based architectures as practical tools for real-time smart waste management systems.

##### Pre-Trained CNN Architectures (Transfer Learning)—Inception and EfficientNet Families

**Figure 18:**
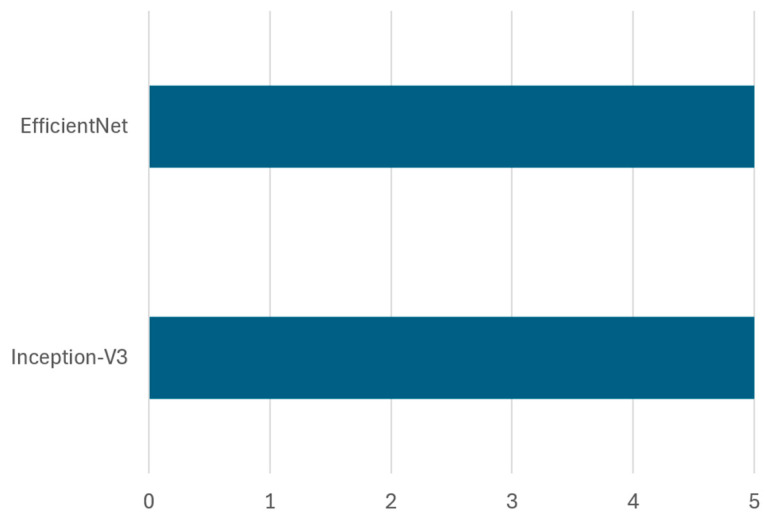
Frequency of Inception and EfficientNet families for waste classification.

Inception and EfficientNet are advanced CNN architectures that have demonstrated strong capability in processing complex image features. Inception models, such as Inception-V3, utilize asymmetric convolutions and multi-scale feature extraction, making them well suited for diverse waste categorization tasks. In contrast, EfficientNet models use compound scaling to balance model depth, width, and resolution, offering high classification accuracy while maintaining computational efficiency. Figure 18 illustrates the implementation frequency of these architectures in waste classification research.

Implementation Trends and Dataset Coverage: As illustrated in Figure 18, both Inception-V3 and EfficientNet have been implemented in five studies each, indicating balanced interest among researchers. These models have been evaluated on benchmark datasets such as TrashNet, Kaggle garbage, TACO, Open Litter Map, OrgalidWaste, and several custom-built datasets. Table 12 summarizes their dataset splits, accuracy outcomes, and cited references.

Performance of Inception-V3: Inception-V3 has demonstrated high performance when applied to well-structured and curated datasets. For instance, Ref. [50] utilized Inception-V3 alongside other pre-trained CNNs, achieving 95.71% accuracy on the garbage classification dataset. Similarly, Ref. [39] applied Inception-V3 for office garbage classification, obtaining 95.33% accuracy, demonstrating its effectiveness in structured waste categories. The high performance of Inception-V3 in these studies highlights its strong feature extraction capabilities, making it suitable for classification tasks involving well-defined waste types. However, performance discrepancies were observed across different datasets. For example, Ref. [45] reported a significantly lower accuracy of 52.83% using Inception-V3 on the Kaggle organic and recyclable waste dataset, indicating that dataset complexity and quality significantly impact model effectiveness. Similarly, Ref. [46] reported only 69.94% accuracy when using Inception-V3 for organic and solid waste classification, further reinforcing that dataset diversity and preprocessing techniques influence classification outcomes.

Effectiveness of EfficientNet Variants: EfficientNet models consistently demonstrated superior performance across multiple studies. This was evident in [76], where EfficientNet-B3 with transfer learning on TrashNet achieved 97% accuracy, showcasing its efficiency in capturing fine-grained features in complex waste images. Similarly, Ref. [88] introduced the ScrapNet dataset and tested EfficientNet-B3, achieving 92.87% accuracy, reinforcing EfficientNet’s adaptability across different waste classification tasks.

EfficientNet in IoT and embedded waste systems: EfficientNet has also been evaluated in real-time waste classification and IoT-based applications. Ref. [63] combined EfficientNet CNN with IoT components, integrating Raspberry Pi, cameras, and ultrasonic sensors for automated waste sorting, achieving 92% accuracy. This indicates EfficientNet’s suitability for embedded systems and smart waste management applications. Similarly, Ref. [90] implemented EfficientNet-B2 for waste detection in natural and urban environments, achieving 75% accuracy, suggesting that EfficientNet’s performance may vary in unstructured settings where waste objects lack uniformity.

In [84], a comparative study tested multiple CNN transfer learning models on TrashNet and reported 87% accuracy with EfficientNet-B7, reinforcing that deeper versions of EfficientNet can enhance results under transfer learning. Similarly, Refs. [70,76] also applied EfficientNet on the TrashNet dataset, achieving accuracies of 87% and 97%, respectively. Further, Ref. [77] combined EfficientNet with a custom CNN model and achieved 97% accuracy, highlighting the potential of hybrid approaches that integrate EfficientNet with other deep learning models. Overall, EfficientNet models demonstrate superior accuracy, adaptability, and efficiency compared to Inception-V3. While both Inception-V3 and EfficientNet have shown strong results, EfficientNet generally outperforms in terms of classification accuracy, adaptability to edge devices, and integration with IoT-based systems. These capabilities make EfficientNet architectures well suited for real-time smart waste classification and highlight their growing relevance for practical deployments.

##### Object Detection Models for Waste Classification

**Figure 19:**
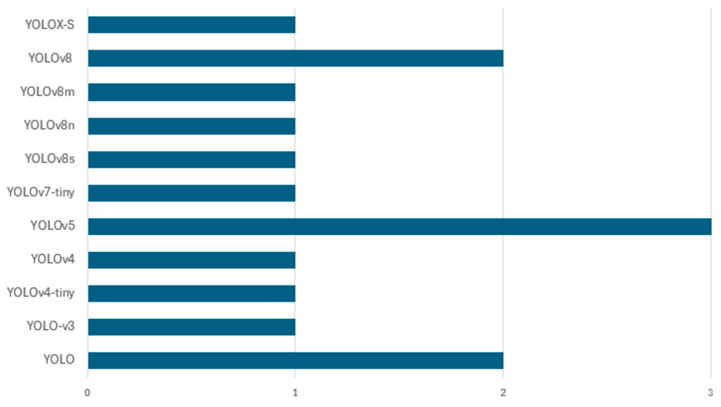
Frequency of object detection models for waste classification.

Object detection models, particularly those based on the YOLO (You Only Look Once) family, have shown remarkable efficiency in real-time waste detection and classification tasks. Unlike traditional CNN models that focus solely on classification, the YOLO-based architecture simultaneously detects and classifies waste objects, making them highly suitable for smart waste management systems, robotics, and IoT-based waste monitoring systems. Figure 19 illustrates the implementation frequency of different YOLO variants in waste classification research.

Implementation Trends and Dataset Coverage: The analysis highlights a broad exploration of YOLO architecture. Among them, YOLOv5 emerged as the most adopted variant with three implementations, followed by YOLO and YOLOv8, each appearing in two studies. Other versions, including YOLOv3, YOLOv4, YOLOv4-tiny, YOLOv7-tiny, YOLOv8s, YOLOv8n, YOLOv8m, and YOLOX-S, were explored in one study each. These models were evaluated across various datasets such as TrashNet, Kaggle, SWAD, TACO, Roboflow, and other custom datasets. Table 13 provides a summary of these implementations.

Performance of YOLO and YOLOv3 Models: The original YOLO model introduced a unified architecture that performs object detection and classification in a single forward pass, enabling real-time processing suitable for embedded systems. YOLOv3 advanced this by adopting Darknet-53 as the backbone and improving anchor box clustering and multi-scale detection. These models have shown promising results in early waste classification applications. For example, Ref. [52] applied YOLO on the Sentinel-2 and Kaggle garbage classification datasets, achieving 93% accuracy with an 80:20 train–test split, demonstrating strong generalization capabilities. In contrast, Ref. [87] tested YOLO on a customized dataset and obtained a significantly lower accuracy of 61%, suggesting that the dataset quality and annotation inconsistencies can degrade model performance. YOLOv3 demonstrated further improvement, as seen in [98], where YOLOv3 was implemented on a dataset containing 1000 real-life household garbage images, achieving an 85% accuracy, indicating its effectiveness in handling real-world waste classification tasks. The enhanced feature extraction capability of YOLOv3, along with better anchor box clustering, led to improved classification accuracy over the standard YOLO model.

YOLOv4 and YOLOv4-Tiny: YOLOv4 introduced several innovations, including CSPDarknet53, PANet, and spatial pyramid pooling (SPP), which improved the model’s ability to capture multiscale features while maintaining real-time speed. YOLOv4-tiny offered a compact version optimized for low-resource devices. Ref. [99] implemented both YOLOv4 and YOLOv4-tiny on the TrashNet dataset, achieving 89.59% and 81.84% accuracy, respectively. These results suggest that while YOLOv4 maintains high accuracy for complex tasks, YOLOv4-tiny trades off some precision for computational efficiency, which is valuable in edge deployments.

YOLOv5, known for its modularity, training flexibility, and deployment-friendly design via PyTorch, has become one of the most widely adopted object detection models. It incorporates improvements such as auto-learning bounding box anchors and mosaic augmentation. Ref. [78] applied YOLOv5 to the Huawei Garbage Classification dataset, achieving an accuracy of 93%, while Ref. [91] reported a significantly higher accuracy of 97.3% using the MMTrash dataset. The superior performance in [91] can be attributed to dataset diversity and fine-tuned hyperparameters, including adaptive learning rate scheduling and enhanced augmentation techniques. However, Ref. [79] applied YOLOv5 to the TACO dataset and reported only 73.5% accuracy, demonstrating the model’s limitations when trained on less diverse or sparsely annotated datasets

YOLOv7 and YOLOv7-Tiny: YOLOv7 and YOLOv7-tiny models introduced further refinements in detection accuracy, computational efficiency, and adaptability. YOLOv7 integrates features like E-ELAN and deeper supervision for improved feature fusion and accuracy, while YOLOv7-tiny is a lightweight alternative designed for fast inference. Ref. [123] implemented YOLOv7-tiny on the WasteInNet dataset, achieving 86.8% accuracy, reinforcing that even lightweight versions can offer strong classification performance while being computationally efficient for edge AI applications.

YOLOv8 is the latest generation in the YOLO family, integrating improvements such as decoupled head structures, spatial attention, and advanced augmentation strategies. It includes variants optimized for different needs: YOLOv8s (small), YOLOv8m (medium), and YOLOv8n (nano). Ref. [100] tested YOLOv8s and YOLOv8m on the TrashNet dataset, both achieving 91.25% accuracy, while YOLOv8n achieved a slightly lower 88.86%. This consistent performance underscores YOLOv8’s robust detection ability. In addition, Ref. [101] implemented YOLOv8 on the SWAD and UAVVaste datasets for aerial surveillance tasks and reported 85.9% accuracy, highlighting its utility for drone-based waste monitoring. Refs. [124,125] reported 97.7% accuracy using YOLOv8 on the Roboflow dataset for solid waste detection, suggesting that proper data preprocessing, augmentation, and fine-tuning significantly impact model performance.

Beyond waste classification, lightweight models like YOLOv5, YOLOv8, and EfficientNet have also been widely studied in other domains such as sustainable agriculture and smart infrastructure, further reinforcing their value for real-time use in constrained environments [124,125].

YOLOX-S is an anchor-free variant that uses decoupled heads for classification and regression, aiming to simplify training and improve generalization. Ref. [92] implemented YOLOX-S on the Trash-Z dataset and additional public datasets (TrashNet, Kaggle, AquaTrash), reporting 85.02% accuracy. Although slightly behind YOLOv8, these results show YOLOX-S to be a competitive and flexible architecture for waste classification, especially when combined with proper tuning and dataset expansion. YOLO-based models—from early versions to the latest iterations like YOLOv8—have evolved to provide an optimal balance between accuracy, speed, and deployment flexibility. Models like YOLOv5, YOLOv8, and YOLOX-S have shown high classification accuracy and adaptability for real-time waste monitoring, UAV-based surveillance, and IoT integration, affirming their growing role in intelligent waste management systems.

##### Specialized CNN Architectures for Waste Classification

Traditional CNN architectures, such as AlexNet and SqueezeNet, have played a pivotal role in early waste classification research due to their lightweight structure, computational efficiency, and strong feature extraction capabilities. These models have been widely applied in studies leveraging transfer learning and data augmentation, especially when operating under limited-resource constraints. Figure 19 illustrates the implementation frequency of these specialized CNN models in waste classification research.

**Figure 20:**
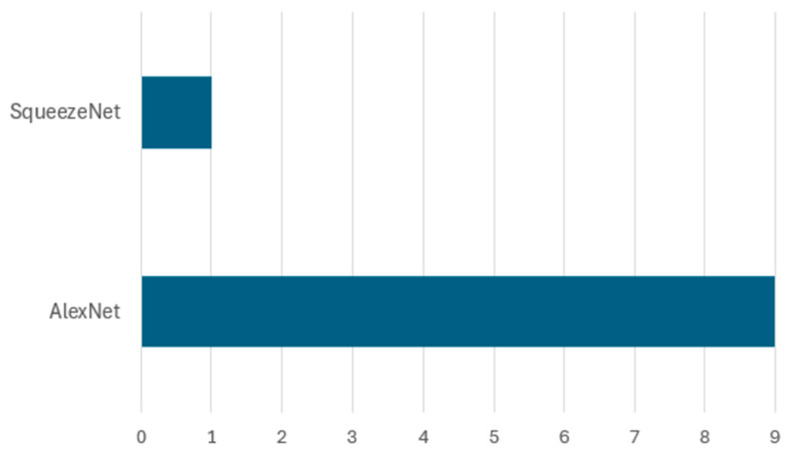
Frequency of specialized CNN models for waste classification.

Implementation Trends and Dataset Use: Figure 20 illustrates that AlexNet was the most commonly adopted architecture, appearing in nine studies, while SqueezeNet was used in a single study. These models were evaluated on datasets such as TrashNet, Kaggle, WaDaBa, and several customized datasets. Table 14 summarizes their dataset usage, breakdowns, achieved accuracy, and methodologies.

Performance of AlexNet in Waste Classification: AlexNet has been widely utilized due to its simple layered architecture, which allows for effective feature extraction with manageable computational cost. However, its performance is influenced by dataset characteristics, augmentation strategies, and training configurations. For example, Refs. [85,121] implemented AlexNet on the WaDaBa dataset, achieving an accuracy of 80.08%, suggesting that AlexNet performs moderately well but struggles with dataset complexity and lighting variations. In contrast, Ref. [82] applied AlexNet within a hybrid transfer learning framework, incorporating Bi-LSTM and Bayesian hyperparameter tuning on the TrashNet dataset, achieving a higher accuracy of 90.26%. This indicates that combining AlexNet with other deep learning techniques enhances classification performance.

AlexNet’s successful integration into real-time embedded systems further reinforces its practical value. In [61], the model was trained on a custom dataset combining TrashNet and other sources, achieving 98.27% accuracy, demonstrating its robustness in classifying biodegradable and non-biodegradable waste. Likewise, Ref. [71] compared AlexNet with DenseNet-121 and SqueezeNet-V1.0, achieving an accuracy of 92.56% on the Kaggle dataset. This suggests that while AlexNet performs well, deeper architectures like DenseNet can offer better classification accuracy when trained on well-structured datasets.

Comparison with Custom CNN Architectures: Several studies have modified or replaced AlexNet with custom CNNs to reduce complexity or optimize for specific scenarios. In [109], a 23-layer AlexNet and a 15-layer custom CNN for plastic waste classification was implemented based on WaDaBa dataset, achieving 99.23% and 97.43% accuracy, respectively. The results indicate that, while deeper networks like 23-layer AlexNet offer superior performance, lightweight models are more efficient for real-time applications on edge devices like Raspberry Pi. Similarly, Ref. [127] designed a 15-layer CNN and compared it with AlexNet for household waste classification, reporting 99.92% accuracy on 120 × 120 images and 91.72% on 227 × 227 images, whereas AlexNet achieved 96.41% and 99.23% on the same resolutions, respectively. The findings suggest that custom architectures optimized for specific image resolutions can enhance the classification performance while reducing computational overhead.

Feature fusion has further improved the effectiveness of AlexNet-based waste classification models. For instance, Ref. [108] introduced a double fusion approach, integrating feature-level and score-level fusion using AlexNet, ResNet101, and GoogleNet on a large-scale waste dataset, achieving 96.2% accuracy. The results highlight that ensemble methods combining multiple deep networks significantly enhance classification robustness, particularly for diverse waste datasets.

SqueezeNet, a lightweight CNN architecture, has also been employed for waste classification on mobile and embedded systems. Ref. [50] compared SqueezeNet-V1.0 with AlexNet and DenseNet-121 on the Kaggle dataset, achieving 91.50% accuracy. While SqueezeNet was outperformed by DenseNet-121 (94.15%), its low parameter count and computational efficiency make it a strong candidate for real-time waste classification in constrained environments.

##### Waste Classification with Deep Learning (Specialized CNN Architectures for Waste Classification)

Encoder–decoder architectures, including autoencoders and CNN-based SegNet models, have also been employed for waste classification and image segmentation. These models utilize an encoding step to extract essential features and a decoding step to reconstruct and classify waste images. Table 15 provides an overview of recent research contributions utilizing encoder–decoder models.

Autoencoder-based models excel in feature extraction by compressing and reconstructing images, as seen in [103], where an autoencoder trained on a merged dataset achieved 81% accuracy. While useful for unsupervised learning, autoencoders generally underperform CNN-based models due to limited robustness in handling complex waste variations. SegNet, a CNN encoder–decoder model, demonstrated superior segmentation accuracy (82.95% IoU in [104]) on the TrashNet dataset. The study highlighted the impact of kernel size and learning rate tuning, with a 5 × 5 kernel size and 45 epochs yielding optimal performance. Unlike autoencoders, SegNet retains spatial information, making it better suited for real-time waste sorting and classification. Overall, autoencoders are valuable for pretraining in label-scarce scenarios, while SegNet provides higher classification and segmentation accuracy, making it ideal for practical waste sorting applications.

**Figure 21:**
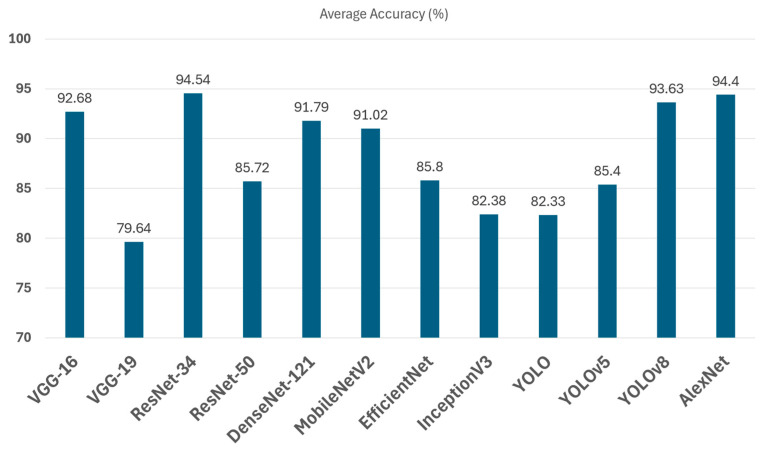
Comparison of average classification accuracy across CNN architectures used in AI-based waste classification.

To synthesize the comparative performance of different CNN architectures, we compared the mean classification accuracy of all CNN architectures discussed in the preceding subsections—ranging from Basic CNN and DCNN Models to the YOLO Family (Object Detection Models). Figure 21 presents the average classification accuracy of the most commonly used models in waste classification. The results show that ResNet-34, AlexNet, YOLOv8, and VGG-16 rank among the top-performing architectures, each achieving average accuracy rates exceeding 92%, with ResNet-34 leading at 94.54%. In contrast, lighter architectures like InceptionV3 and certain YOLO variants showed lower average performance, although they are often preferred for their real-time deployment capabilities. These findings emphasize that while deeper models generally offer superior accuracy, their suitability depends on the target deployment context, computational constraints, and dataset quality.

### 3.3. Hybrid Approaches for Waste Classification

While Section 3.1 and Section 3.2 have discussed publicly available datasets and the standalone applications of machine learning and deep learning techniques, this section focuses on hybrid approaches that integrate multiple AI methods to improve performance. Hybrid models aim to leverage the strengths of different algorithms—such as combining CNNs for feature extraction with classifiers like SVM or random forest—to overcome the limitations associated with individual techniques.

Hybrid models have proven effective in enhancing classification accuracy, adaptability, and computational efficiency, particularly when applied with heterogeneous and real-world waste datasets. Hybrid deep learning approaches integrate multiple deep learning techniques to improve the efficiency and accuracy of waste classification systems. Typically, these models combine convolutional neural networks (CNNs) with other architectures such as artificial neural networks (ANNs), recurrent networks, transformer-based models, and optimization techniques to leverage the strengths of each method. Hybrid models often employ feature fusion strategies, transfer learning, and metaheuristic optimization techniques to refine their classification capabilities. By integrating different models, hybrid approaches enhance feature extraction, improve the classification accuracy, and reduce computational complexity. Table 16 shows the different proposed hybrid approaches for waste classification.

Several studies have demonstrated the effectiveness of hybrid deep learning models for waste classification. A novel three-stage classification framework using a parallel depth-wise separable CNN (DP-CNN) combined with an ensemble extreme learning machine (En-ELM) was introduced in [40]. The model first classifies waste into biodegradable and non-biodegradable categories, then refines the classification into nine types, and finally differentiates among 36 specific waste categories. This hierarchical classification structure significantly reduces computational costs while maintaining high accuracy, with performance levels reaching 96% in the first stage and 85.25% in the final classification stage.

Another approach integrating a fully convolutional network (FCN) with a deep belief network (DBN) and modified rat swarm optimization (MRSO) was proposed in [59]. The FCN is used for object detection, DBN performs hierarchical classification, and MRSO optimizes network hyperparameters, allowing the model to achieve an impressive 99.2% accuracy on a kitchen waste dataset. This study demonstrates how combining CNN architectures with optimization algorithms can significantly improve the classification performance compared to standalone CNNs such as ResNet50 and VGG16.

The combination of CNN and ANN has also been explored for improving waste classification accuracy. A study in [44] introduced a hybrid CNN-ANN model trained on the Kaggle trash dataset, achieving 97% accuracy. The CNN extracts deep feature representations, while the ANN is used for classification, outperforming traditional machine learning models like support vector machines (SVMs) and k-nearest neighbors (kNNs). Similarly, another study [128] combined CNN with graph long short-term memory (GLSTM) to improve real-time municipal waste classification. By integrating CNN for initial object detection and GLSTM for temporal sequence prediction, the system enhances real-time processing capabilities, achieving 98% accuracy.

Optimization-based hybrid models have also been proposed to improve classification accuracy. The fractional horse herd gas optimization (FrHHGO)-based shepherd CNN (ShCNN) [129] integrates multiple optimization techniques for e-waste classification, achieving 95% accuracy. The model utilizes an IoT-cloud platform for e-waste image collection and feature extraction methods such as gray-level co-occurrence matrix (GLCM), histogram of oriented gradients (HOG), and local directional ternary patterns (LDTP). Similarly, the use of two EfficientNet models combined with a custom CNN [117] achieved 97% accuracy by integrating image hashing and augmentation techniques to improve the dataset quality and address overfitting. Hybrid models have also been designed for real-time waste classification and object detection. The multilayer perceptron (MLP) combined with a multilayer CNN (ML-CNN) in [60] was developed for smart cities, using a camera-based conveyor belt waste classification system. The model classifies waste into metal and non-metal categories with 99% accuracy. A multilayer hybrid convolution neural network (MLH-CNN) proposed in [130] further optimized the CNN architectures using smaller kernels and batch normalization techniques, achieving 93% accuracy.

Another study [80] combined single-shot detectors (SSDs) with regional proposal networks (RPNs) to enhance the object detection for real-time waste identification, reporting 97.63% accuracy for SSD and 95.76% for faster R-CNN. Incorporating multiple CNN models into a single hybrid architecture has also proven effective. GCDN-Net [93], a combination of DenseNet201 and Inception-v3, achieved 95.77% accuracy for single-label classification and an mAP of 0.69 for multi-label classification, demonstrating the advantage of combining CNNs with different feature extraction capabilities. RWC-Net [94], another hybrid model combining DenseNet201 and MobileNetV2, was designed for recyclable waste classification, outperforming traditional CNN models with an accuracy of 95.01%. Additionally, a Bi-LSTM model integrated with CNN-based transfer learning [82] improved the classification for the TrashNet dataset, achieving 96.67% accuracy.

Transformers have also been introduced into waste classification frameworks, showcasing improvements over CNN-based models. The customized vision transformer (ViT-WM), combined with CNN and RNN [95], achieved 98.17% accuracy by leveraging transfer learning on a large-scale dataset consisting of TrashNet, Kaggle Waste Dataset, and Google Images. The transformer-based model outperformed both CNN and RNN models in classification accuracy, highlighting the potential of transformer architectures for automated waste management.

Furthermore, a recent study [96] introduced ECCDN-Net, a hybrid architecture that synergistically fuses Densenet201 and ResNet18 with auxiliary outputs, achieving 96.1% accuracy on a customized image dataset. By leveraging the dense feature extraction of DenseNet201 and the residual learning of ResNet18, the model significantly enhances classification precision in binary waste categories (organic vs. recyclable), demonstrating its potential for real-world deployment in automated waste sorting systems.

Similarly, the DWSD dataset [42] evaluated multiple hybrid semantic segmentation models—DeepLabv3+, UNet, PSPNet, and FPNet—for pixel-level waste classification, where DeepLabv3+ achieved the highest accuracy of 89.39%, demonstrating the effectiveness of CNN-based segmentation architectures in complex, densely cluttered waste scenarios.

In summary, hybrid deep learning models have demonstrated significant improvements in waste classification accuracy by integrating CNNs with other architectures, optimization techniques, and real-time processing methods. The use of CNN-ANN combinations, metaheuristic optimizations, and transformer-based models has led to robust classification frameworks capable of efficiently handling large-scale waste datasets. While Section 3.1, Section 3.2 and Section 3.3 have explored the taxonomy, applications, and effectiveness of ML, DL, and hybrid models in AI-based waste classification, it is equally important to examine how these approaches perform in real-world contexts. Section 3.4 extends this analysis by presenting actual deployment case studies, prototype systems, and TRL (technology readiness level) assessments, thereby bridging the gap between academic research and practical implementation. These real-world applications not only validate the feasibility of AI-powered systems but also uncover new challenges encountered during field deployment. Building on this, the next section presents a forward-looking discussion of critical gaps, open issues, and emerging research directions to ensure the scalability, reliability, and sustainability of intelligent waste classification systems in operational environments.

### 3.4. Real-World Implementations and TRL-Level Analysis

Several studies have progressed beyond theoretical exploration and simulations to demonstrate the real-world or near-deployment implementations of AI-powered waste classification systems. These implementations often involve embedded devices, IoT integration, and real-time processing capabilities, and can be evaluated through the lens of technology readiness levels (TRLs). Table 17 summarizes the implementation stage and TRL of the selected works.

For instance, Ref. [49] deployed a VGG-16 model on a Raspberry Pi equipped with a camera and sensors for smart bin classification, achieving 98.15% accuracy, which corresponds to TRL 6–7—a demonstration in a relevant environment. Similarly, Ref. [62] implemented SSD MobileNetV2 Quantized using TensorFlow Lite for real-time detection on embedded devices, achieving 80% accuracy, indicating TRL 5–6 suitability for edge-based pilot systems. A hybrid model combining CNN with Bi-LSTM was deployed in [82] for real-time trash classification using the TrashNet dataset, achieving 96.67% accuracy and indicating maturity at TRL 6. Likewise, Ref. [63] reported the successful deployment of EfficientNet integrated with Raspberry Pi, ultrasonic sensors, and cameras for smart sorting bins, reaching 92% accuracy, again positioning it at TRL 6. The study in [87] combined ResNet-50 with YOLO for a real-time object detection pipeline, yielding 96.83% accuracy in a relevant environment, estimated at TRL 6–7. Meanwhile, Ref. [81] applied YOLOv8 on UAV-based solid waste detection using the Roboflow dataset, achieving 97.7% accuracy, demonstrating performance in a simulated operational setting—estimated at TRL 5–6. These works collectively illustrate how state-of-the-art deep learning and hybrid models are now transitioning from lab environments to operational prototypes, signaling progress toward TRL 7 and TRL 8, especially for smart waste segregation, IoT applications, and autonomous monitoring systems.

As the adoption of AI in waste management moves toward practical deployment, it is also essential to consider the ongoing limitations and research gaps that hinder full-scale implementation. The following section discusses key challenges, unresolved issues, and future research directions critical for developing scalable, trustworthy, and efficient AI-based waste classification systems.

## 4. Challenges, Limitations, and Future Directions

**Figure 22:**
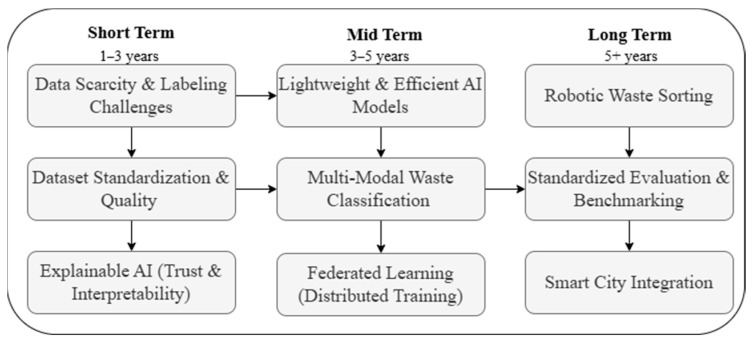
Proposed roadmap for AI-based waste classification: phased challenges and future directions.

AI-driven waste classification is a rapidly evolving research area with significant potential to improve waste management systems. However, transitioning from experimental models to practical deployment necessitates addressing a set of interlinked challenges. Figure 22 presents a phased roadmap, organizing these challenges and opportunities into three types, namely the short-term, mid-term, and long-term categories, based on technical dependencies and implementation readiness. The roadmap highlights how early-stage issues—such as data scarcity and standardization—directly influence mid-term advances like lightweight models, federated learning, and multi-modal classification. In the long term, resolving these foundational issues can pave the way for large-scale, sustainable deployments such as robotic waste sorting, smart city integration, and globally standardized AI benchmarks. Each of the following subsections elaborates on these directions, aiming to bridge technical innovation with sustainable and scalable AI applications in waste management.

### 4.1. Data Scarcity and Standardization Challenges

The primary challenge that waste classification faces in AI-based waste classification is the lack of large-scale, diverse, and standardized datasets, which limit model generalization. Many studies rely on small and domain-specific datasets which limit their applicability to broader waste categories [59,130]. Although data augmentation techniques and synthetic data generation have been proposed to compensate for these limitations, their effectiveness in real-world deployment remains limited. Resolving data scarcity and improving dataset standardization are thus recognized as short-term (1–3-year) priorities, forming a critical foundation for advancing more complex AI models and achieving reliable performance in practical applications.

Several benchmark datasets, such as TrashNet and WaDaBa [31], have served as foundations for early research, studies by [79,88] continue to highlight persistent issues with dataset quality and annotation consistency. Research on the WaDaBa dataset by [85,109] demonstrates that even specialized datasets fall short in capturing real-world waste diversity. Similarly, recent work by [69] using the TrashBox dataset, achieved 97.4% accuracy but identified significant challenges in maintaining annotation quality across diverse waste types. Work on the Trash-Z dataset by [92] showed that, while combining multiple public datasets may help mitigate data scarcity, the lack of unified taxonomy and standard labeling remains a substantial barrier to effective model training.

In addition, Refs. [59,93] emphasize the urgent need for publicly available datasets that include broader waste categories, diverse lighting conditions, and varying environmental contexts. Future research must focus on developing more comprehensive and standardized datasets that better represent these real-world waste scenarios. Furthermore, this review considered only English-language publications from major databases such as IEEE Xplore, Scopus, and Web of Science. Expanding future reviews to include multilingual databases and regional repositories could yield a more globally representative synthesis of the literature.

### 4.2. Addressing Waste Complexity in Real-World Environments

Real-world waste classification presents unique challenges due to occlusions, poor lighting, mixed materials, and complex backgrounds that current AI solutions have not yet fully addressed [128,129]. These complexities significantly affect model reliability when transitioning from controlled datasets to deployment in unpredictable environments. Researchers must therefore prioritize enhancing dataset diversity to better reflect real-world conditions and focus on developing AI models capable of generalizing across different waste textures, occlusion levels, and environmental contexts.

Addressing these real-world variations is identified as a short-term (1–3-year) priority, essential for improving the robustness of AI models in uncontrolled deployment environments. By advancing models that can operate reliably under diverse and noisy conditions, we not only improve system reliability but also directly contribute to broader sustainability goals. Enhanced classification robustness reduces sorting errors, increases recycling efficiency, and minimizes the environmental impact of misclassification. These improvements support sustainable development goals (SDGs), particularly SDG 11 (sustainable cities and communities) and SDG 12 (responsible consumption and production), by enabling smarter, more resilient urban waste management systems and promoting efficient resource recovery. Recent empirical studies highlight the performance gaps when AI models encounter environmental complexity. For instance, Ref. [79] used the TACO dataset and achieved only 73.5% accuracy, underscoring the challenges of handling naturally occurring waste imagery. The work by [86] on the Taco trash dataset demonstrated promising results with 97% accuracy, but still reported significant challenges under inconsistent lighting and occlusion. Similarly, research by [101] involving UAV-based aerial waste detection achieved just 85.9% accuracy, indicating further room for improvement in handling high-altitude, real-world variability. Future systems must better tackle these complex, unpredictable conditions to ensure reliable waste classification in practical deployments.

### 4.3. Lightweight and Efficient AI Models

The increasing shift toward edge computing and IoT-based smart waste systems has made the development of lightweight and computationally efficient AI models a critical necessity. Traditional deep learning models, though highly accurate, are computationally intensive and often unsuitable for real-time waste classification on embedded devices. This has led to growing interest in model compression techniques, pruning strategies, and neural architecture search (NAS) to reduce energy and memory consumption without sacrificing performance.

Designing lightweight and efficient AI models is recognized as a mid-term (3–5 year) research objective, closely linked to progress in data quality, interpretability, and deployment validation. Optimizing models for constrained environments enables low-latency processing in real-world applications such as automated waste bins, drones, and mobile robots. These improvements also align with sustainability principles by reducing energy demand, extending device lifespan, and enabling decentralized waste monitoring in resource-limited settings. Several studies have demonstrated promising results in this area. For example, Refs. [59,131] highlight the use of lightweight architectures and NAS for developing energy-efficient models. In [62], an SSD MobileNetV2 quantized model achieved 80% accuracy while maintaining real-time performance on embedded devices, showing the practicality of quantized models. Similarly, Ref. [73] demonstrated 90.7% accuracy using an optimized MobileNetV2, and Ref. [122] achieved 98% accuracy with a compact model but noted trade-offs in terms of hardware constraints and memory usage. These examples collectively highlight the importance of achieving a balance between model efficiency and classification accuracy. Moreover, architectures like MobileNet and TinyML have shown potential for real-time deployment, but many existing studies primarily report accuracy without evaluating models across other critical dimensions such as latency, energy consumption, and inference time [16]. Future work should establish more comprehensive benchmarking protocols that facilitate robust comparisons across CNN, YOLO, MobileNet, and hybrid architectures. Such benchmarking can enable more informed model selection for practical use in waste classification and support scalable, eco-efficient deployments in real-world smart environments.

### 4.4. Explainable AI (XAI) for Trustworthy Classification

While deep learning models have shown impressive performance in waste classification, their opaque decision-making processes present a significant barrier to trust and adoption. Most of these models operate as black-box systems, providing little insight into why a particular classification was made. This lack of interpretability can hinder human validation, limit transparency, and reduce acceptance in critical waste management scenarios, especially in regulated or safety-sensitive environments [77,130]. Explainable AI (XAI) addresses these concerns by making model predictions interpretable and verifiable. Techniques such as Grad-CAM, SHAP, and LIME generate visual or feature-level explanations that allow users to understand which parts of an image contributed to the classification decision. For example, Ref. [61] incorporated Grad-CAM visualizations to provide insights into feature activation patterns during classification. However, achieving both high model accuracy and interpretability remains an open challenge. In [61], a real-time embedded waste classification system reached 98.15% accuracy but lacked any explainability mechanism, limiting its potential for human oversight and debugging in deployment scenarios. As AI systems continue to move closer to real-time field deployment, explainability will become increasingly important to support transparency, safety, and accountability. Integrating XAI into AI-driven waste classification pipelines can help build trust among stakeholders, facilitate human-in-the-loop validation, and support ethical and regulatory compliance. Explainable AI is thus identified as a mid-term (3–5 year) research priority. Its integration will be essential not only for improving system transparency but also for fostering greater human trust and acceptance—key enablers of successful adoption in practical smart waste management environments.

### 4.5. Multi-Modal Waste Classification

Current AI models primarily rely on RGB image classification, but integrating multi-modal approaches such as hyperspectral imaging, chemical sensing, and infrared scanning could significantly enhance classification accuracy [94,128]. Multi-modal learning enables AI models to extract complementary features, improving the differentiation of similar waste types. Research like [94] demonstrates that combining visual and spectral data leads to more robust waste classification. Research by [59] demonstrated that combining visual data with other sensor inputs achieved 99.2% accuracy through their FCN-DBN hybrid approach. Similarly, Ref. [128]’s implementation of CNN with GLSTM showed that temporal data integration could improve a real-time classification performance to 98%. The work by [40] on the TriCascade waste image dataset revealed that hierarchical classification using multiple data streams could achieve up to 96% accuracy in the first stage of classification. Future research should focus on fusion techniques for multi-modal inputs to enhance the classification performance across various waste categories. Multi-modal waste classification is a mid-term (3–5 years) target that relies on earlier improvements in dataset diversity, sensor integration, and standardized data pipelines.

### 4.6. Self-Supervised and Few-Shot Learning for Waste Classification

Self-supervised learning (SSL) and few-shot learning (FSL) are promising approaches for addressing the heavy reliance on large-scale labeled datasets in AI-based waste classification. Unlike supervised learning, which requires manually annotated data, SSL enables models to learn useful feature representations from unlabeled data through pretext tasks. Similarly, FSL allows a model to quickly adapt to new waste categories using only a small number of labeled examples. These techniques are particularly relevant for waste classification, where labeling is resource-intensive due to the diversity and ambiguity of waste items. Recent studies support the effectiveness of using transfer learning and hybrid architectures as precursors to SSL and FSL advancements. For instance, Ref. [59] showed that transfer learning significantly boosts classification accuracy, laying the foundation for the future adaptation of SSL and FSL. Likewise, Ref. [76] achieved 97% accuracy using EfficientNet-B3, while Ref. [77] demonstrated that hybrid models combining EfficientNet with custom CNNs can attain a similarly high performance. Additionally, Ref. [82] employed Bi-LSTM with transfer learning and achieved 96.67% accuracy on the TrashNet dataset, further validating the strength of these adaptive learning strategies.

Given these developments, self-supervised and few-shot learning approaches represent a mid-term (3–5 years) research priority, where further innovation is expected to reduce dependence on large-scale annotated datasets. Future research should focus on developing domain-specific self-supervised frameworks and prototypical networks tailored for heterogeneous waste types. Advancing these methods can significantly improve generalization in real-world settings, particularly where data collection and annotation are constrained. Such techniques are likely to play a pivotal role in enhancing the scalability and adaptability of AI-powered waste classification systems.

### 4.7. Federated Learning for Decentralized Waste Classification

Federated learning (FL) is emerging as a viable solution to the privacy and scalability limitations of centralized AI models in waste classification systems. Unlike traditional cloud-based approaches where data must be uploaded to a central server, FL allows AI models to be trained locally on distributed devices or edge nodes. This decentralized training process helps maintain user privacy, reduce communication overhead, and mitigate the risk of data breaches, which are critical considerations in municipal and industrial waste management scenarios [130,132].

Recent studies have demonstrated the promise of FL in collaborative environments. For instance, Ref. [132] suggests that federated learning can enable cooperative AI training across multiple waste collection centers, facilitating knowledge sharing without compromising sensitive information. Moreover, Ref. [95] implemented the ViT-WM architecture and achieved 98.17% accuracy, indicating the potential effectiveness of distributed learning models when trained across diverse data sources. Similarly, Ref. [93] introduced the GCDN-Net model, which integrates multiple CNN architectures and achieved 95.77% accuracy on single-label classification tasks. Although not explicitly federated, such modular architectures could be adapted for FL settings to support distributed yet coordinated learning. Federated learning represents a mid-term (3–5 years) research direction in AI-based waste classification. Its successful adoption will depend on parallel advances in standardizing datasets, improving device-level efficiency, and incorporating privacy-preserving techniques such as differential privacy and secure aggregation. As AI systems are increasingly deployed at the edge—within smart bins, recycling units, and municipal infrastructures—FL can play a pivotal role in enabling secure, scalable, and real-time waste classification while aligning with sustainable smart city initiatives.

### 4.8. AI-Driven Robotic Waste Sorting

Integrating artificial intelligence with robotic systems represents a pivotal advancement in automating waste sorting processes. AI-powered robotic systems, equipped with vision-based detection and grasping capabilities, offer a promising alternative to manual sorting in recycling facilities and waste treatment plants. These systems can identify, classify, and sort waste items based on material properties, shape, and recyclability, thereby increasing efficiency, reducing human exposure to hazardous materials, and supporting sustainable waste management operations [60,80]. Recent works have demonstrated the feasibility of such integrations. For instance, Ref. [123] employed YOLOv7-tiny for real-time object detection, achieving 86.8% accuracy, highlighting its applicability for robotic vision tasks. In a similar vein, Ref. [81] implemented YOLOv8 for solid waste detection, reporting a 97.7% accuracy rate, underscoring its potential for deployment in automated robotic sorting pipelines. The HHGO-based CNN model proposed by [129] supports the notion that synergizing deep learning with robotics substantially improves sorting accuracy and operational reliability in complex waste scenarios.

AI-driven robotic waste sorting is considered a long-term (5+ years) research direction, requiring substantial progress in the maturity of detection models, real-time inferencing capabilities, sensor calibration, and deployment-ready AI infrastructure. Nevertheless, this direction aligns closely with global sustainability goals and smart industry initiatives by minimizing human intervention and optimizing waste segregation at scale.

These future directions highlight the significant opportunities and challenges in advancing AI-based waste classification systems. Success in these areas could substantially improve the efficiency and reliability of automated waste management systems while addressing critical environmental challenges. In addition to these technical trajectories, future research could also explore broader implications within entrepreneurial ecosystems, particularly in relation to smart infrastructure and sustainable innovation. Literature from the fields of innovation and entrepreneurship suggests the need for stronger integration between technological development and commercialization pathways [133]. While this aspect lies outside the scope of the present review, it represents a promising direction for future interdisciplinary collaboration aimed at translating AI-based waste solutions into scalable, real-world applications.

## 5. Conclusions

This systematic review has provided a comprehensive examination of machine learning and deep learning techniques in waste classification, dividing the approaches into four main categories: supervised learning, unsupervised learning, deep learning, and hybrid learning. Through the analysis of 97 research articles from reputable sources such as Web of Science, IEEE, and Science Direct, this study has revealed the significant advancements and current state of AI-driven waste classification. Our review has compiled over fifteen benchmarked datasets relevant to waste classification, offering valuable resources for researchers in this domain. However, the analysis also highlights that many of these datasets suffer from limitations: such as class imbalance, annotation inconsistencies, and lack of environmental diversity that can lead to inconsistent performance when applying the same AI technique across different datasets, ultimately affecting model generalization and reliability. Moreover, the comparative analysis of various AI techniques shows that deep learning models (72.67%) and hybrid approaches (10.47%) dominate the current research landscape, while traditional supervised learning (15.1%) and unsupervised learning (1.74%) still play meaningful roles in specific contexts. The study also highlights that while CNN-based architectures demonstrate strong performance, especially when enhanced with transfer learning, emerging hybrid models that combine multiple computational approaches often achieve the highest accuracy rates, frequently exceeding 95%. This review also identifies several persistent challenges and limitations in existing approaches, including data imbalance, environmental variability, and computational resource constraints, especially for real-time and edge deployment scenarios. To address these issues, a structured research roadmap has been proposed, categorizing future directions into short-, mid-, and long-term priorities. This roadmap emphasizes improvements in classification accuracy, model efficiency, and the sustainable deployment of AI in smart waste management systems. The comprehensive findings presented in this review enhance the current understanding of AI applications in waste management and serve as a valuable reference for future research exploring innovative, efficient, and scalable waste classification frameworks.

## Figures and Tables

**Table 1:** Identified survey and review papers.

References	Year of Publication	Covered Years	Survey Type	Waste Classification Methods	Machine Learning Methods	Deep Learning Methods	Hybrid Models	Datasets Discussed	Accuracy Comparisons	Future Directions
[10]	2021	2000–2020	Review	✔	✔	✔	✘	✘	✘	✘
[14]	2021	2016–2021	Review	✔	✘	✘	✘	✘	✘	✔
[15]	2021	2000–2019	Review	✔	✘	✘	✘	✘	✘	✘
[16]	2022	2000–2022	Survey	✔	✘	✔	✘	✔	✔	✔
[17]	2023	2017–2023	SLR	✔	✔	✔	✘	✔	✘	✔
[18]	2023	2019–2023	Review	✔	✘	✘	✘	✘	✘	✔
[19]	2023	1965–2022	Review	✔	✘	✘	✘	✘	✘	✔
[20]	2024	2000–2023	SLR	✔	✔	✔	✘	✘	✔	✔
This article		2020–2025	SLR	✔	✔	✔	✔	✔	✔	✔

**Table 2:** Inclusion and exclusion criteria.

Inclusion Criteria	Exclusion Criteria
Studies specifically addressing machine learning in waste management.	Non-empirical studies like theoretical analyses, opinions, and reviews.
Studies have been published within the last 5 years.	Studies not focusing on machine learning in waste management.
Peer-reviewed articles in reputable journals or proceedings.	Publications older than ten years.
Studies available in full text for comprehensive evaluation.	Articles published in languages other than English.
Studies conducted and published in English	Studies only available in abstract form or without complete texts.
Strong baseline papers selected for review.	Papers with limited relevance.
Only reproducible papers whose works can be extended.	Papers which are general discussions.

**Table 3:** Available datasets and their types of images.

Reference	Dataset Name	No. of Images	Type of Images
[24]	TrashNet Dataset	2400	Glass, paper, metal, plastic, cardboard, trash
[26]	CompostNet Dataset	2751	Paper, cardboard, metal, glass, plastic, trash, compost, food waste, landfill waste
[27]	WasteRL Dataset	57,000	Organic waste, recyclables, hazardous waste, other wastes (annotated with bounding boxes)
[28]	Kaggle Waste Classification Dataset	22,500	Organic waste, recyclable waste
[29]	GINI Dataset	2561	Trash (956 images directly related to garbage; remaining from Bing image search)
[30]	TACO Dataset	1500	Plastic, glass, metal, paper, cardboard, trash (plastic bags, cigarette butts, bottles, cans, other common litter)
[31]	WaDaBa Dataset	4000	Plastic waste items (photographed under different conditions of lighting and angle)
[32]	Open Litter Map Dataset	>100,000	Glass, paper, metal, plastic, cardboard, trash (various types of litter in natural environments)
[33]	Domestic Trash Dataset	>9000	Plastic, metal, glass, paper, cardboard, trash (plastic cups, batteries, razors, plastic bags)
[34]	TrashBox Dataset	17,785	Glass, metal, plastic, paper, cardboard, e-waste, medical waste
[35]	GIGO: Garbage In, Garbage Out Dataset	25,000	Cardboard, plastic, trash (bulky waste, garbage bags, cardboard, litter)
[36]	ZeroWaste Dataset	4661	Glass, paper, metal, plastic, cardboard, trash
[37]	VN Trash Dataset	>13,000	Plastic, metal, glass, paper, cardboard, trash (aluminum cans, carton, foam box, milk box, clear plastic cup, PET bottle, other trash)
[38]	Baidu Garbage Classification Dataset	17,690	Glass, paper, metal, plastic, cardboard, trash (recyclable garbage, food waste, hazardous garbage, other garbage; 158 sub-categories)
[12]	NWNU-TRASH Dataset	18,911	Waste glass, waste fabric, waste paper, waste plastic, and waste metal
[39]	Custom Dataset	2313	Glass, paper, metal, plastic, cardboard, trash (office garbage: cans, bottles, milk boxes, paper cups, batteries)
[40]	TriCascade WasteImage	35,264	Green waste, recyclable waste, glass, metal, polymer (petroleum-based), leather and fabric, medical waste, E-waste, hazardous waste
[41]	TrashNeXt dataset	23,625	Cardboard, E-waste, foam rubber, glass, medical waste, metal, paper, plastic, organic
[42]	DWSD (Dense Waste Segmentation Dataset)	784	Plastic container, plastic bottle, Thermocol, metal bottle, plastic–cardboard, glass, Thermocol–plate, plastic, paper, plastic cup, paper cup, aluminum foil, cloth, nylon

**Table 4:** Model training platforms of corresponding studies.

Platform	References
TensorFlow	[39,40,41,45,46,49,51,52,61,62,63,64,65,66,67,68,69,70,71,72,73,74,75,76,77,78,79,80]
PyTorch	[12,44,63,73,81,82,83,84,85,86,87,88,89,90,91,92,93,94,95,96]
Google Colab	[42,44,46,51,62,63,67,70,74,76,84,86,93,94,97,98,99,100,101]
Keras	[40,44,46,48,49,51,53,64,66,68,70,71,82,102,103,104]
MATLAB	[57,58,103,104,105,106,107,108,109]
Scikit-learn	[110,111]
Jupyter	[41,45]
PyCharm	[40,60]

**Table 5:** Waste classification with supervised learning.

Technique/Model	Dataset/Usage	Dataset Breakdown (Train–Test or Train–Val–Test%)	Accuracy	Reference
SVM	TrashNet dataset	80:20	85%	[54]
	TrashNet dataset	85:15	65%	[55]
	IoT sensor data from residential waste bins	70:30	78%	[56]
	Thung Yang dataset	70:30	63%	[57]
	Custom dataset of 15,000 images	80:20	89.6%	[58]
	Kaggle dataset of waste photos	80:20	94.8%	[59]
	Kaggle Trash dataset	80:20	84%	[60]
	Waste images from Kaggle and Google	85:15	80%	[61]
	TrashNet and local garbage datasets	80:20	62.5%	[62]
KNN	IoT sensor data from residential waste bins	70:30	77%	[56]
	Construction site waste dataset	70:30	88.51%	[63]
	Custom dataset of 15,000 images	80:20	87.3%	[58]
	Kaggle trash dataset	80:20	94.1%	[60]
Random forest	TrashNet dataset	80:20	55%	[54]
	IoT sensor data from residential waste bins	70:30	85.29%	[56]
	Kaggle trash dataset	80:20	95.2%	[60]
	TrashNet and local garbage datasets	80:20	72%	[62]
Decision tree	TrashNet dataset	80:20	65%	[54]
	IoT sensor data from residential waste bins	70:30	84.1%	[56]
	Construction site waste dataset	70:30	88.32%	[63]
ANN	Municipal waste data	-	98%	[64]
	Waste characterization data from Johannesburg	80:20	96.1%	[65]
Naïve Bayes	IoT sensor data from residential waste bins	70:30	84.1%	[56]
	Kaggle trash dataset	80:20	51.2%	[60]
Logistic regression	IoT sensor data from residential waste bins	70:30	80%	[56]

**Table 6:** Waste classification with unsupervised learning.

Technique/ Model	Dataset/Usage	Key Finings	Reference
K-means clustering	Multi-objective solid waste dataset	Employed unsupervised learning to identify patterns and classifications in solid waste without pre-labeled data.	[54]
	GIS data for potential MSW landfill site evaluation	Effective use of GIS and clustering for evaluating and prioritizing landfill sites based on multiple criteria.	[116]
	Data on 1139 fuel samples including HHV	Introduced HOM CS for better fuel classification based on physical properties. Effective in optimizing fuel use for energy conversion.	[111]
Voronoi graph theory	Data from Beijing’s urban garbage collection and transportation system	Efficiency improved from 74.9% to 95.6%.	[106]
Fuzzy C-means clustering with SCA	1000 records of simulated waste data based on oil spill classifications	Developed a cluster-based technique to classify oily waste types from marine oil spill operations, improving waste management strategies.	[117]

**Table 7:** Waste classification with supervised learning using CNN and DCNN models.

Technique/Model	Dataset/Usage	Dataset Breakdown (Train–Test or Train–Val–Test)	Accuracy	Reference
CNN	Custom dataset of 15,000 images	80:20	95.4%	[118]
	Customized dataset with Kaggle trash dataset	80:10:10	89.88%	[64]
	Kaggle garbage classification dataset	70:15:15	94.40%	[45]
	Thung Yang dataset	70:13:17	22%	[24]
	Kaggle garbage classification dataset	80:20	83%	[43]
	TrashNet dataset	80:20	90%	[97]
	Customized images augmented with TACO dataset	80:10:10	92%	[55]
	OrgalidWaste dataset	70:20:10	80.31%	[74]
	TrashNet dataset	90:10	76%	[75]
	Kaggle garbage classification dataset	80:20	96%	[76]
	TrashNet dataset	80:20	92.7%	[62]
Custom CNN	Kaggle garbage classification dataset	80:20	97.16%	[77]
DCNN	Customized images	70:30	70%	[107]
	Kaggle garbage classification dataset	70:30	93%	[119]
	Customized dataset	75:25	98%	[66]
	Customized dataset	85.7:14.3	90–97%	[58]

**Table 8:** Waste classification with VGG family.

Technique/Model	Dataset/Usage	Dataset Breakdown (Train–Test or Train–Val–Test)	Accuracy	Reference
VGG-16	TrashNet dataset	70:15:15	87.25	[82]
	Kaggle garbage classification dataset	70:20:10	92%	[48]
	Sentinel-2, Kaggle garbage classification dataset	80:20	93%	[52]
	Kaggle garbage classification dataset	90:10	98%	[49]
	Kaggle garbage classification dataset	80:20	87.5%	[50]
	Kaggle garbage classification dataset	80:20	93.37%	[43]
	OrgalidWaste dataset	70:20:10	88.42%	[46]
	Kaggle garbage classification dataset	70:15:15	93.49%	[51]
	Customized dataset	70:10:20	95.60%	[83]
	Customized images from TrashNet dataset	85.7:14.3	98.15%	[61]
VGG-19	Kaggle garbage classification dataset	70:15:15	56%	[45]
	Kaggle garbage classification dataset	70:20:10	91%	[48]
	OrgalidWaste dataset	70:20:10	86.38%	[46]
	Customized dataset	80:20	85.17%	[108]

**Table 9:** Waste classification with ResNet family.

Technique/Model	Dataset/Usage	Dataset Breakdown (Train–Test or Train–Val–Test%)	Accuracy	Reference
ResNet-18	TrashNet dataset	90:10	95.8%	[56]
ResNet-34	TrashNet dataset	70:20:10	91.50%	[82]
	TrashNet dataset	70:30	94.64%	[84]
	VNTrash, TrashNet dataset	Not Mentioned	96.27%	[67]
	Kaggle garbage classification dataset	80:20	91.8%	[43]
	Customized dataset	70:20:10	98.5%	[53]
ResNet-50	TrashNet dataset	70:20:10	92.45%	[82]
	Kaggle garbage classification dataset	70:20:10	95%	[48]
	WaDaBa dataset	80:20	85.34%	[109]
	Customized dataset	70:30	50.92%	[68]
	Kaggle garbage classification dataset	70:15:15	66.67%	[45]
	Customized dataset	80:20	94.3%	[50]
	Customized images from TrashNet dataset	85.7:14.3	97.9%	[61]
	WaDaBa dataset	80:20	85.5%	[85]
	Taco trash dataset	95:5	97%	[86]
	Customized dataset	70:10:20	96.6%	[83]
	Customized dataset	80:20	96.8%	[87]
	OrgalidWaste dataset	70:20:10	50.28%	[46]
	Kaggle garbage classification dataset	70:15:15	93.02%	[51]
	Customized dataset	80:20	96%	[112]
	TrashNet dataset	70:30	92.24%	[84]
	ScrapNet dataset	80:20	83.11%	[88]
	Customized dataset	80:20	84.1%	[89]
ResNet-101	ScrapNet dataset	80:20	80.5%	[88]
	customized	80:20	87.76%	[108]
ResNet-152	TrashNet dataset	80:20	70.7%	[69]
	ScrapNet dataset	80:20	79.11%	[88]
ResNeXt	WaDaBa dataset	80:20	87.44%	[85,121]

**Table 10:** Waste classification with DenseNet family.

Technique/ Model	Dataset/Usage	Dataset Breakdown	Accuracy	Reference
sDenseNet121	WaDaBa dataset	80:20	85.58%	[121]
	WaDaBa dataset	80:20	85.5%	[85]
	Customized Dataset	70:30	91%	[68]
	TrashNet dataset	90:10	94%	[102]
	TrashNet dataset	70:30	94.4%	[84]
	TrashNet dataset	85:15	93.3%	[70]
	VNTrash, TrashNet	-	96.4%	[67]
	Kaggle garbage classification dataset	70:20:10	94.1%	[71]
DenseNet161	TrashBox dataset	80:20	97.4%	[69]
DenseNet169	TrashNet dataset	70:30	95.6%	[84]
	NWNU-TRASH dataset	70:30	82.8%	[12]
DenseNet201	TrashNet and local garbage datasets	80:20	96%	[120]

**Table 11:** Waste classification with MobileNet family.

Technique/ Model	Dataset/Usage	Dataset Breakdown	Accuracy	Reference
MobileNetV2	Kaggle garbage classification dataset	70:20:10	93%	[48]
	Kaggle garbage classification dataset	80:20	96.9%	[50]
	VNTrash, TrashNet	-	96.2%	[67]
	WaDaBa dataset	80:20	87.3%	[121]
	Customized dataset	80:10:10	90%	[68]
	WaDaBa dataset	80:20	87.3%	[85]
	TrashNet dataset	85:15	93%	[70]
	Customized dataset	80:10:10	83%	[72]
	Customized dataset	70:30	80%	[62]
	Huawei Cloud datasets	-	90.7%	[73]
	Custom bag classification dataset	64:16:14	98%	[122]
	BDWaste dataset	80:20	96.8%	[74]
MobileNetV3	TrashBox dataset	80:20	85.9%	[69]
	Huawei garbage classification dataset	83.3:16.7	92.6%	[75]

**Table 12:** Inception and EfficientNet families for waste classification.

Technique/ Model	Dataset/Usage	Dataset Breakdown	Accuracy	Reference
Inception-V3	Kaggle garbage classification dataset	80:20	95.7%	[50]
	OrgalidWaste dataset	70:20:10	69.9%	[64]
	TrashNet + custom images	85.7:14.3	98.15%	[61]
	Kaggle’s organic and recyclable waste dataset	70:15:15	52.83%	[45]
	Custom dataset	80:20	95.33%	[39]
EfficientNet	TrashNet dataset	70:30	97%	[76]
	Custom dataset	70:20:10	92%	[63]
	TrashNet dataset	70:30	98.02%	[84]
	Kaggle garbage classification dataset	80:20	35.92%	[50]
	TACO, Open Litter Map, TrashNet	85:15	87%	[90]
	ScrapNet dataset (combination of TrashNet, OpenRecycle, TACO)	80:20	92.8%	[88]
	TrashNet dataset	85:15	87%	[70]
	OrgalidWaste dataset	60:20:20	97%	[77]

**Table 13:** YOLO-based families for waste classification.

Technique/ Model	Dataset/Usage	Dataset Breakdown	Accuracy	Reference
YOLO	Sentinel-2, Kaggle garbage classification dataset	80:20	93%	[52]
	Customized dataset	80:20	61%	[87]
YOLO-v3	1000 real-life household garbage images	90:10	85%	[98]
YOLOv4-tiny	TrashNet dataset	80:20	81.84%	[99]
YOLOv4	TrashNet dataset	80:20	89.59%	[99]
YOLOv5	Huawei garbage classification	83.3:16.7	93%	[78]
	TACO dataset	70:20:10	73.5%	[79]
	MMTrash dataset	70:30	97.3%	[91]
YOLOv7-tiny	WasteInNet dataset	70:30	86.8%	[123]
YOLOv8s	TrashNet dataset	70:15:15	91.25%	[100]
YOLOv8n	TrashNet dataset	70:15:15	88.86%	[100]
YOLOv8m	TrashNet dataset	70:15:15	91.25%	[100]
YOLOv8	SWAD + UAVVaste datasets	75:15:10	85.9%	[101]
	Roboflow dataset for solid waste detection	78:9:13	97.7%	[81]
YOLOX-S	Trash-Z dataset + public datasets (TrashNet, Kaggle, AquaTrash)	90:10	85.02%	[92]

**Table 14:** Specialized CNN models (AlexNet and SqueezeNet) for waste classification.

Technique/Model	Dataset/Usage	Dataset Breakdown	Accuracy	Reference
AlexNet	WaDaBa dataset	80:20	80.08%	[85,121]
	TrashNet dataset	70:15:15	90.26%	[82]
	Kaggle garbage classification dataset	70:30	99.20%	[126]
	Custom dataset augmented with images from TrashNet and other sources	85.7:14.3%	98.27%	[61]
	Kaggle garbage classification dataset	70:20:10	92.56%	[71]
	WaDaBa dataset	90:10	99.23%	[109]
	Customized	80:20	99.2%	[108]
	Customized plastic waste dataset	-	96.41%	[127]
SqueezeNet	Kaggle garbage classification dataset	70:20:10	91.50%	[71]

**Table 15:** Specialized CNN models (autoencoders) for waste classification.

Technique/ Model	Dataset/Usage	Dataset Breakdown	Accuracy	Reference
Autoencoder	TrashNet dataset + Kaggle garbage classification dataset	80:20	81%	[103]
	TrashNet dataset	80:20	82.9%	[104]

**Table 16:** Hybrid models for waste classification.

Technique/Model	Dataset/Usage	Dataset Breakdown	Accuracy	Reference
Parallel lightweight depth-wise separable CNN (DP-CNN) + ensemble extreme learning machine (En-ELM)	TriCascade WasteImage dataset	80:10:10	Stage 1: 96% Stage 2: 91% Stage 3: 85.2%	[40]
Fully convolutional network (FCN) + deep belief network (DBN) + modified rat swarm optimization (MRSO)	Images of kitchen waste from Kaggle garbage classification dataset	70:30	99.2%	[59]
Custom CNN + ANN (artificial neural network)	Kaggle trash dataset	80:20	97%	[44]
CNN + graph-long short-term memory (GLSTM)	Customized garbage image dataset	80:20	98%	[128]
Fractional horse herd gas optimization-based shepherd CNN (FrHHGO-based ShCNN)	Gofile E-waste dataset	70:30	95%	[129]
EfficientNet models + Custom CNN	OrgalidWaste dataset	60:20:20	97%	[77]
Multilayer perceptron + multilayer convolutional neural network (ML-CNN),	Real-time environment + customized images of waste	90:10	99%	[60]
Multilayer hybrid convolution neural network (MLH-CNN)	TrashNet database	90:10	93%	[130]
CNN (single-shot detectors (SSD) and regional proposal networks (RPNs))	TrashNet database	90:10	97.63% (SSD), 95.76% (Faster R-CNN)	[80]
GCDN-Net (combination of DenseNet201 + Inception-v3)	GIGO dataset	70:10:20	75.01%	[93]
RWC-Net (combination of DenseNet201 + MobileNetV2)	TrashNet database	70:20:10	95%	[94]
Bi-LSTM + transfer learning (CNN-based models)	TrashNet database	70:15:15	96.67%	[82]
Parallel lightweight depth-wise separable CNN (DP-CNN) + Ensemble extreme learning machine (En-ELM)	TriCascade waste image dataset	80:10:10	96%	[40]
GMC-MobileNetV3 (Improved MobileNetV3 with CBAM, Mish activation function, and global average pooling)	Customized dataset	70:20:10	96.55%	[131]
ResNet and Custom CNN models	TrashNet database	67:33	88.66%	[132]
Customized vision transformer (ViT-WM) + CNN + RNN	TrashNet dataset + Kaggle waste dataset + Google Images	80:20	98.1%	[95]
ECCDN-Net (Densenet201 + Resnet18 + auxiliary outputs)	Customized dataset	70:20:10	96.1%	[96]
DeepLabv3+, UNet, PSPNet, FPNet	DWSD dataset	82:18	DeepLabv3+: 89.39%, UNet: 88.99%, PSPNet: 83.80%, FPNet: 81.33%	[42]

**Table 17:** Real-world waste classification implementations and estimated TRLs.

Reference	Model Used	Reported Accuracy	Implementation Notes	Estimated TRL
[61]	VGG-16	98.15%	Deployed on Raspberry Pi with camera	TRL 6–7
[62]	SSD MobileNetV2 Quantized	80%	Real-time waste detection on embedded devices with TensorFlow Lite	TRL 5–6
[82]	Bi-LSTM + Transfer Learning	96.67%	Trash classification using hybrid CNN and Bi-LSTM in real-time	TRL 6
[63]	EfficientNet	92%	Deployed using Raspberry Pi, cameras, and ultrasonic sensors	TRL 6
[87]	ResNet-50 + YOLO	96.83%	Real-time object detection pipeline	TRL 6–7
[81]	YOLOv8	97.7%	Waste detection for UAV-based system	TRL 5–6

## Data Availability

All data analyzed in this study are derived from previously published studies, which are referenced in the manuscript.

## Abbreviations

The following abbreviations are used in this manuscript:

ML	Machine Learning
DL	Deep Learning
IoT	Internet of Things
RFID	Radio-Frequency Identification
WasteRL	Waste Reinforcement Learning
EDA	Exploratory Data Analysis
CNN	Convolutional Neural Network
SVM	Support Vector Machine
ANN	Artificial Neural Network
KNN	K-Nearest Neighbor
RNN	Recurrent Neural Network
MTLA	Multi-task Learning Architecture
GAN	Generative Adversarial Network
SCA	Subtractive Clustering Algorithm
YOLO	You Only Look Once
MGD	Multi-Look Ground Range Detected
W2R	Waste Recognition-Retrieval
RegM	Recognition Metric
IoU	Intersection over Union
MHS	Multi-Hybrid System
MLP	Multilayer Perceptron
LSTM	Long Short-Term Memory
ELM	Extreme Learning Machine
PCA	Principal Component Analysis
DRL	Deep Reinforcement Learning
KAN	Knowledge-Augmented Networks
GNN	Graph Neural Networks
MSME	Micro-, Small-, and Medium-sized Enterprise
SLR	Systematic Literature Review
PRISMA	Preferred Reporting Items for Systematic Reviews and Meta-Analyses
GCDN	Garbage Classifier Deep Neural
RWC	Recyclable Waste Classification
RevM	Retrieval Metric
DQLN	Deep Q-Learning Network
DSCR	Deep Spatial Contextual Representation
HOG	Histogram of Oriented Gradients
SIFT	Scale-Invariant Feature Transform
SURF	Speeded Up Robust Features
PLS-DA	Partial Least Squares Discriminant Analysis
LoRa	Long Range
GIS	Geographic Information System
mAP	Mean Average Precision
AR	Average Recall
